# Gut microbiota mediates anxiety-like behaviors induced by chronic infection of *Toxoplasma gondii* in mice

**DOI:** 10.1080/19490976.2024.2391535

**Published:** 2024-08-25

**Authors:** Xiaotong Luo, Xiaoying Yang, Shimin Tan, Yongsheng Zhang, Yunqiu Liu, Xiaokang Tian, Yingting Huang, Yuying Zhou, Cheng He, Kun Yin, Daxiang Xu, Xiangyang Li, Fenfen Sun, Renxian Tang, Jianping Cao, Kuiyang Zheng, Yinghua Yu, Wei Pan

**Affiliations:** a Jiangsu Key Laboratory of Immunity and Metabolism, Jiangsu International Key Laboratory of Immunity and Metabolism, Department of Pathogen Biology and Immunology, Xuzhou Medical University, Xuzhou, Jiangsu, China; b The Second Clinical Medical College, Xuzhou Medical University, Xuzhou, Jiangsu, China; c National Demonstration Center for Experimental Basic Medical Science Education, Xuzhou Medical University, Xuzhou, Jiangsu, China; d Shandong Institute of Parasitic Diseases, Shandong First Medical University & Shandong Academy of Medical Sciences, Jining, Shandong, China; e National Institute of Parasitic Diseases, Chinese Center for Disease Control and Prevention (Chinese Center for Tropical Diseases Research), NHC Key Laboratory of Parasite and Vector Biology, WHO Collaborating Centre for Tropical Diseases, Shanghai, China

**Keywords:** *Toxoplasma gondii*, anxiety, gut microbiota, fecal microbiota transplantation, succinate, Diethyl butylmalonate

## Abstract

**Background:**

Chronic infection with the neurotropic parasite *Toxoplasma gondii (T. gondii)* can cause anxiety and gut microbiota dysbiosis in hosts. However, the potential role of gut microbiota in anxiety induced by the parasite remains unclear.

**Methods:**

C57BL/6J mice were infected with 10 cysts of *T. gondii.* Antibiotic depletion of gut microbiota and fecal microbiota transplantation experiments were utilized to investigate the causal relationship between gut microbiota and anxiety. Anxiety-like behaviors were examined by the elevated plus maze test and the open field test; blood, feces, colon and amygdala were collected to evaluate the profiles of serum endotoxin (Lipopolysaccharide, LPS) and serotonin (5-hydroxytryptamine, 5-HT), gut microbiota composition, metabolomics, global transcriptome and neuroinflammation in the amygdala. Furthermore, the effects of Diethyl butylmalonate (DBM, an inhibitor of mitochondrial succinate transporter, which causes the accumulation of endogenous succinate) on the disorders of the gut-brain axis were evaluated.

**Results:**

Here, we found that *T. gondii* chronic infection induced anxiety-like behaviors and disturbed the composition of the gut microbiota in mice. In the amygdala, *T. gondii* infection triggered the microglial activation and neuroinflammation. In the colon, *T. gondii* infection caused the intestinal dyshomeostasis including elevated colonic inflammation, enhanced bacterial endotoxin translocation to blood and compromised intestinal barrier. In the serum, *T. gondii* infection increased the LPS levels and decreased the 5-HT levels. Interestingly, antibiotics ablation of gut microbiota alleviated the anxiety-like behaviors induced by *T.* *gondii* infection. More importantly, transplantation of the fecal microbiota from *T. gondii*-infected mice resulted in anxiety and the transcriptomic alteration in the amygdala of the antibiotic-pretreated mice. Notably, the decreased abundance of succinate-producing bacteria and the decreased production of succinate were observed in the feces of the *T. gondii*-infected mice. Moreover, DBM administration ameliorated the anxiety and gut barrier impairment induced by *T.* *gondii* infection.

**Conclusions:**

The present study uncovers a novel role of gut microbiota in mediating the anxiety-like behaviors induced by chronic *T. gondii* infection. Moreover, we show that DBM supplementation has a beneficial effect on anxiety. Overall, these findings provide new insights into the treatment of *T. gondii*-related mental disorders.

## Introduction

Anxiety is one of the highly prevalent mental disorders,^1^ which has a serious impact on people’s life quality and contributes to the exacerbation of various diseases, including inflammatory bowel disease.^2^ *Toxoplasma gondii* (*T. gondii*) is a neurotropic parasite, approximately infecting one-third of the world population.^3^ The chronic infection of the parasite is mainly characterized by the existence of cysts in the brains of hosts, although several cysts are also found in other organs (*e.g.*, the muscle and liver).^4–6^ For a long time, the effects of chronic infection on mental disorders have been neglected due to no obvious clinical performance. However, the emerging evidence in recent years indicates that chronic infection of *T. gondii* may be a potential etiology of anxiety. For example, long-term infection of the parasite has been reported to induce anxiety-like behaviors in rodent models.^7–10^ In line with these findings, higher titers of *T. gondii* IgG are related to anxiety and depression in women during pregnancy.^11^ Moreover, individuals who are positive for *T. gondii* IgG have over twice the odds of having generalized anxiety disorder compared to seronegative individuals.^12^

The amygdala is a vital brain region responsible for anxiety.^13^ The declined levels of neurotransmitters (*e.g*., 5-HT) are the molecular indicator for anxiety.^14^ It is reported that the cysts of *T. gondii* can be detected in the amygdala with high intensity, resulting in the activation of microglia and neuroinflammation.^15^ Several studies have demonstrated that pro-inflammatory cytokines including TNF-α and IL-1β can induce anxiety-like behaviors in mice, and contrarily, suppressing these cytokines can relieve the condition.^16,17^ Moreover, the expression of these pro-inflammatory cytokines is also significantly up-regulated in patients with anxiety-related disorders.^18^ Overall, these findings suggest that neuroinflammation in the amygdala is the important neuropathological basis for anxiety.

Gut microbiota and its metabolites can regulate neuroinflammation and anxiety *via* the gut-brain axis.^19^ For example, exposure to microbial metabolite 4-ethylphenyl sulfate (4EPS) can induce anxiety-like behaviors in mice.^20^ Moreover, LPS produced by most Gram-negative bacteria can compromise the blood-brain barrier, thereby inducing neuroinflammation *via* activating microglia and consequently, anxiety-like behaviors.^21–23^ In addition, succinate-producing bacteria including *Lachnospiraceae* and *Akkermansia*, are reported to alleviate anxiety-like behaviors by maintaining gut health.^24–27^ Thus, probiotics targeting gut microbiota can effectively improve anxiety in both animal and human cohorts.^28,29^ In recent years, there has been increasing evidence that long-term *T. gondii* infection induces the alteration of gut microbiota in mice.^30–32^ However, it is still unknown whether microbiota dysbiosis is associated with the anxiety induced by chronic *T. gondii* infection.

The present study showed that gut microbiota dysbiosis contributes to the anxiety-like behaviors induced by *T. gondii* chronic infection. Interestingly, the decreased abundance of succinate-producing bacteria and the lower levels of succinate were observed in the feces of *T. gondii*-infected mice. Notably, the administration of DBM, an inhibitor of the mitochondrial succinate transporter, which causes the accumulation of endogenous succinate,^33,34^ attenuated the anxiety-like behaviors. Overall, these findings uncover a crucial role of gut microbiota in mediating *T. gondii*-induced anxiety, and provide a novel insight for developing gut microbiota-based therapies against *T. gondii*-related mental disorders.

## Methods

### Animals, parasites

C57BL/6J mice (7 weeks old) were obtained from Beijing Vital River Laboratory Animal Technology Co. Ltd. (Beijing, China) and were bred in the Experimental Animal Center of Xuzhou Medical University. All mice were housed in an air-conditioned room at 24°C with a 12 h dark/light cycle and permitted free access to food (Catalogue No. 1002, Pizhou Xiaohe Technology Development Co., Ltd, Xuzhou, China) and autoclaved tap water. After arriving in the new environment, the mice were acclimatized for one week before being used for experiments. TgCtWh6, a strain of *T. gondii* that often causes chronic infection and prevails in China,^35^ was gifted by the laboratory of Professor Jilong Shen.

### *T.* *gondii* infection

The mice were randomly divided into two groups (*n* = 8 mice/group): (I) Mice received the phosphate buffer saline (PBS) by gavage as a control (Con) group; (II) Mice received the cysts of TgCtWh6 by gavage (10 cysts for each mouse) as Tg group. The exact process of infection was carried out as previously described.^36^ The anxiety-like behavior tests were performed four weeks post infection. Mice were sacrificed with CO_2_ three days after behavior testing. Blood, feces, colon, and amygdala samples were collected for further analyses.

### Broad-spectrum antibiotic intervention experiment

Broad-spectrum antibiotics were used to ablate the gut microbiota of mice as previously reported.^37^ In brief, the mice were given drinking water containing neomycin (1 g/L), ampicillin (1 g/L), metronidazole (1 g/L), and vancomycin (0.25 g/L) (all purchased from BBI LIFE SCIENCE Co. Ltd Company) for two weeks. Then they were administered *T. gondii* infection, and continuously given this drinking until the end of the experiment. Mice were sacrificed with CO_2_ three days after behavioral testing. The amygdala was collected for further analysis.

### Fecal microbiota transplantation

For the fecal microbiota transplantation (FMT) experiment, the recipient mice were first given drinking water containing antibiotic cocktail to eliminate the gut microbiota, and then transplanted fecal microbiota from Con and Tg-infected mice. In detail, mice first received a combination of wide-spectrum antibiotics in their drinking water containing neomycin (1 g/L), ampicillin (1 g/L), metronidazole (1 g/L), and vancomycin (0.25 g/L), which was renewed per three days for successive three weeks, and then switched to normal drinking water for three days to eliminate the pharmacological effect of antibiotics. Subsequently, disinfected cages were daily prepared to pool feces from Con and Tg-infected mice, and 100 mg feces (about 5–6 fecal pellets) were then put into new sterilized tubes. The fresh feces were mixed with sterile PBS at a dilution ratio of 100 mg/1000 μL. After being filtered by the filter with a pore size of 70 μm, filtering liquor was centrifuged at 4°C for 5 min, and continued to take suspension centrifugal washing to gain the final bacterial suspension, which was diluted with an equal volume of sterile PBS (approximately containing10^11^CFU/L flora) before oral gavage to recipient mice (10 mL/kg) individually for seven successive days.^38^ It took two weeks to colonize the fecal microbiome before the behavioral tests.^37^

### Diethyl butylmalonate (DBM) administration

To demonstrate the role of succinate in the anxiety induced by *T. gondii* infection, the DBM was administrated in the *T. gondii-*infected mice. In brief, mice were randomly divided into four groups. In the Con+Veh group, mice received PBS as vehicle control. In the Con+DBM group, control mice received 40 mg/kg DBM (112038-100 ML, Sigma-Aldrich, St. Louis, USA). In the Tg+Veh group, Tg-infected mice were intraperitoneally injected with PBS. In the Tg+DBM group, Tg-infected mice received the same dose of DBM. DBM administration (intraperitoneal injection, twice per week), started at three days before *T. gondii* infection until the ending of behavioral tests.

### Behavior testing

The elevated plus maze test (EPMT) and the open field test (OFT) were used to measure anxiety-like behaviors in mice.^39–41^ For EPMT, a gray elevated plus maze apparatus was used. The elevated plus maze consists of two open arms and two closed arms which radiated from a central area. The same arms were opposite to each other. Adjacent arms were placed at right angles. In the test, the mice were put in the central area and made them face the open arm to explore the maze for 5 min. The process was recorded by a video camera and the maze was cleaned with 70% ethanol to remove residual odors before each test. The mice with higher anxiety would visit the open arm less often and stay there for a shorter time.

For OFT, a square open-air box was used to do this test. The environment should be quiet and the box should be cleaned with 70% ethanol just like before. Before the test, mice were taken to the room to adapt to the new environment for nearly 1 hour. Mice were habituated for 5 min in the open-air box without recording. Half an hour later, mice were lowered from the center of the box to explore for 10 min. All mouse movements were recorded by a video camera. A less anxious mouse tends to be interested in the central area and shows a desire to explore. While mice with high anxiety tend to walk against the walls of the box or hide in corners.

### 16S rRNA gene sequencing analysis

At the end of the experiment, feces were collected from mice and stored at −80°C for subsequent analysis. Microbial DNA was extracted from the cecal contents of mice using the E.Z.N.A. stool DNA Kit (Omega Bio-tek, Norcross, GA, U.S.). Specific primers with barcode (sequencing joint) were used to amplify the 16S rDNA V3-V4 region. The primer was 341F: CCTACGGGNGGCWGCAG; 806 R: GGACTACHVGGGTATCTAAT. The amplified products were mixed in equal quantities, the sequencing junctions were connected, sequencing libraries were constructed, and then conducted computer sequencing with Illumina. Polymerase chain reaction (PCR) was performed in triplicate 50 μL mixture containing 1.5 μL of each primer (5 μM), 5 μL of 2.5 mM dNTPs, 5 μL of 10 × KOD Buffer, 1 μL of KOD polymerase and 100 ng of template DNA Amplicons extracted from 2% agarose gels. Purification was performed using the AxyPrep DNA Gel Extraction Kit (Axygen Biosciences, Union City, CA, USA) according to the manufacturer’s instructions, and quantification was performed by QuantiFluor-ST (Promega, USA). Purified amplicons were pooled in identical molar and paired-end sequences (2 × 250) on the Illumina platform following the standard protocols. When analyzing, after raw reads were obtained by sequencing, low-quality reads were filtered, and then spliced and filtered to obtain OTU by clustering.

### Transcriptome analysis

Fresh amygdala tissues from *T. gondii*-infected mice and FMT mice were collected for analyzing the global transcriptomic profile. Total RNA was extracted using the Trizol kit (Invitrogen, Carlsbad, CA, USA) according to the manufacturer’s protocol, and mRNA was enriched by Oligo (dT) beads. The enriched mRNA was fragmented into a short fragmentation buffer and reverse transcribed into cDNA. The purified double-stranded cDNA fragments were end-repaired, “A” base added, and ligated to Illumina sequencing adapters. The ligation reaction was purified with the AMPure XP Beads (1.0X). Ligated fragments were selected for size by agarose gel electrophoresis and PCR amplification was performed. The resulting cDNA libraries were sequenced using Illumina Novaseq6000 and differentially expressed mRNAs were identified by Gene Denovo Biotechnology Co. using the DESeq2 program and their relative numbers were reflected by the number of individual gene reads. Genes with a *P*-value <0.05 and |log_2_fold change| >0.58 were identified as significantly differentially expressed genes between the two samples. GO enrichment analysis was performed at Metascape (https://metascape.org/gp/index.html). Only terms with a *p* < 0.05 were considered significant. KEGG pathway enrichment analysis was performed using the KEGG pathway database (http://www.kegg.jp/.) and pathways with *p* < 0.05 were considered significantly different between the two groups.

### Untargeted metabolome

Fecal tissues (100 mg of each sample) were ground separately in liquid nitrogen and the homogenate was resuspended with 500 μL of pre-cooled 80% methanol by vortexing. The samples were incubated on ice for 5 min and then centrifuged at 15,000 g for 20 min at 4°C. A portion of the supernatant was diluted with LC-MS grade water to a final concentration containing 53% methanol. The samples were subsequently transferred to a new Eppendorf tube and then centrifuged at 15,000 g for 20 min at 4°C. Finally, the supernatants were injected into the LC-MS/MS system analysis.

UHPLC-MS/MS analysis was performed using a Vanquish UHPLC system (ThermoFisher, Germany) with an Orbitrap Q ExactiveTM HF-X mass spectrometer (Thermo Fisher, Germany) at Gene Denovo Co., Ltd. (Guangzhou, China). Samples were injected onto a Hypersil Gold column (100 × 2.1 mm, 1.9 μm) using a 17-min linear gradient at a flow rate of 0.2 mL/min. The eluents for the positive polarity mode were eluent A (0.1% FA, water) and eluent B (methanol). The eluents for the negative polarity mode were eluent A (5 mM ammonium acetate, pH 9.0) and eluent B (methanol). The Q Exactive TM HF-X mass spectrometer was run in positive/negative polarity mode. The raw data files generated by UHPLC-MS/MS were processed using Compound Discoverer 3.1 (CD3.1, Thermo Fisher) to perform peak alignment, peak picking, and quantitation for each metabolite. Following that, the intensities of the peaks were normalized to the total spectral intensity. The normalized data was used to predict molecular formulae based on additive ions, molecular ion peaks and fragment ions. These peaks were then matched against mzCloud (https://www.mzcloud.org/), mz Vaultand Mass List database to obtain accurate qualitative and relative quantitative results. Statistical analyses were carried out using the statistical software R (R version *R*-3.4.3), Python (Python 2.7.6 version) and CentOS (CentOS release 6.6).

### Immunofluorescence staining

Colonic ZO-1 staining was utilized to evaluate the integrity of the gut barrier. After sacrificing the mice, the colon tissues were collected and fixed in a 4% paraformaldehyde solution (G1101, Servicebio, China). Five μm colon sections were stained with anti-ZO-1 antibody (GB11195, 1:200, Servicebio) and goat-anti-rabbit CY3 conjugated antibody (GB21303, 1:300, Servicebio), and then counterstained with DAPI (G1012, Servicebio).

After perfusing with cold PBS, the brains of mice were collected and fixed in 4% paraformaldehyde (G1101, Servicebio, China) overnight, and continued to be placed in 30% sucrose water for two days to dehydrate. Frozen brain sections of 20 μm were cut using a cryostat at − 18°C. The brain slices were blocked with 1% BSA for 40 min at 37°C and then incubated with the primary antibody anti-calcium-binding adapter molecule 1 (Iba1, 019-19741, 1:500, Wako) at 4°C overnight. After washing with PBS, the sections were incubated with secondary antibodies at room temperature for 2 h. The secondary antibody goat-anti-rabbit Cy3 conjugated antibody (GB21303, 1:400, Servicebio) was used. Finally, the sections were counterstained with DAPI (G1012, Servicebio) and then imaged with the microscope (OLYMPUS IX51). Image J software was used to quantify Iba-1 immunoreactivity in each field.

### Western blotting

The amygdala was homogenized in ice-cold RIPA lysis buffer including a complete EDTA-free protease inhibitor cocktail. The supernatant was collected, and a BCA assay was used to quantify the protein concentration. Equal amounts of protein were separated by sodium dodecyl sulfate-polyacrylamide gel electrophoresis (SDS-PAGE) and transferred onto polyvinylidene difluoride (PVDF) membranes. The membrane was blocked with 5% non-fat milk at room temperature for 1 h and then incubated overnight at 4°C with primary anti-IL-6 (ab290735, 1:1000, abcam) and β-actin (81115–1-RR, 1:20,000, Proteintech) antibodies. Following 3 washes in Tris Buffered Saline Tween (TBST), the membrane was incubated with HRP-linked anti-rabbit IgG secondary antibody (GB23303, 1:3000, Servicebio) at room temperature for 1 h. After washing 3 times with TBST, the protein bands were detected with Clarity™ ECL western blot substrate (Bio-Rad, 1,705,060) and visualized using the ChemiDoc Touch imaging system (Bio-Rad). The expression of protein in each sample was normalized to β-actin.

### RNA extraction and quantitative (q) real-time polymerase chain reaction

Total RNA was extracted with the RNA isolater total RNA extraction reagent (Vazyme Biotech Co., Ltd, Nanjing, China) from the colon and the amygdala tissues. The concentration of total RNA was confirmed at 260 nm and 280 nm using a spectrophotometer (DU800, Beckman Coulter Inc., Brea, CA, USA). Then, 1 μg of purified RNA was reverse-transcripted to generate cDNA with HiScript II Q RT SuperMix for qPCR (+gDNA wiper) (Vazyme Biotech Co., Ltd, Nanjing, China). The qPCR was performed using ChamQ SYBR qPCR Master Mix (Vazyme Biotech Co., Ltd, Nanjing, China) and assayed on a real-time PCR detection system (Roche, Switzerland). The relative mRNA expression level was determined using the 2^−ΔΔCt^ method with β-actin as the internal reference control. Primer sequences were supplemented in Table S1.

### Lipopolysaccharide (LPS) determination

The LPS levels in the serum of mice were detected using a chromogenic end TAL kit (Limulus assay kit, Cat.18110115, Xiamen Bioendo Technology Co., Ltd, Xiamen, China). The absorbance was measured by a spectrophotometer at 545 nm wavelength, with measurable concentrations ranging from 0.1 to 1.0 EU/ml. All samples for LPS measurements were performed in duplicate.

### 5-HT determination

5-HT levels in the serum were detected using a chromogenic end TAL kit (RXJ99904, RUIXIN BIOTECH, China). The absorbance was measured by spectrophotometer at 450 nm wavelength. All samples for 5-HT measurements were performed in duplicate.

### Succinate determination in serum

Succinate levels were detected by ultra-performance liquid chromatography-tandem mass spectrometry (UPLC-MS/MS). Succinate was extracted by mixing acetonitrile/methanol/water with 10 μL of serum. Targeted analysis of succinate was performed using an ultra-performance LC system coupled to a mass spectrometer (Waters XEVO TQD, USA) with negative electrospray ionization (ESI). Succinate was retained and separated by hydrophilic interactions using a C18 column (Waters ACQUITY UPLC BEHC18 column, 2.1 mm × 100 mm, 1.7 μm). Mobile phase A was water and formic acid, mobile phase B was acetonitrile and formic acid. The peak area was integrated to measure the concentration of succinate. All samples for succinate measurements were performed in duplicate.

### Propionate determination

Feces were collected to determine the propionate levels. Gas chromatography-mass spectrometry (GC-MS) was employed with Helium as the carrier gas at a coincident flow rate of 1 mL/min. The original oven temperature was maintained at 60°C for 5 min upregulated to 250°C at the speed of 10 ℃/min, and held at 250°C for 5 min. The temperatures of the electron impact ion source, transmission line, and front entrance were set at 230, 250, and 280°C, respectively. Data processing was carried out with the Agilent MSD Chemical Station (Agilent).

### Bacterial quantification in feces

To detect the effect of antibiotic cocktails on gut microbiota, the bacterial load was absolutely quantified in the feces of mice following antibiotic treatment or vehicle for three weeks. Total DNA was isolated from known amounts of feces using the QIAamp DNA Stool Mini Kit (Qiagen). DNA was then subjected to quantitative PCR using the QuantiFast SYBR Green PCR Kit (Biorad) with universal 16S rRNA primers (5′-ACTCCTACGGGAGGCAGCAG-3′ and 5′-ATTACCGCGGCTGCTGG-3′) to measure total bacteria number. 16S rRNA gene abundance in fecal samples was determined by comparing the cycle threshold (Ct) values to those of the standard curve. Standard curve was created by extracting and quantifying DNA from a known number of *Escherichia coli*.

### Fluorescein isothiocyanate (FITC)-dextran assay

Intestinal permeability was assessed with an in *vivo* FITC-dextran (FD4; Sigma-Aldrich, St. Louis, USA) permeability assay, as described previously.^42^ Mice fasted for 4 h were gavaged with 0.6 mg/g body weight FITC-dextran (4 kDa) in a 75 mg/mL solution. Plasma was collected 4 h post-gavage feeding. The fluorescence intensity in plasma was measured using a fluorescence spectrophotometer at 485 (excitation) and 528 nm (emission). Serial dilutions of FITC-dextran in PBS were used to calculate a standard curve.

### Cyst burden counting

The brain tissues of mice were homogenized in 1 mL PBS. Then, 10 μL brain suspension was taken and screened under a light microscope (×20 magnification). The cyst number was counted in a blind manner to estimate the total cyst burden in the brain tissue. The counting process for each mouse was repeated at least 3 times.

### Determination of tachyzoites inhibition rate

To evaluate the direct inhibition effect of antibiotics and DBM against *T. gondii*, free tachyzoites from *T. gondii* were cultivated in the presence of antibiotic cocktails (20, 50, 100, 200 μg/mL; neomycin: ampicillin: metronidazole: vancomycin = 4: 4: 4: 1), DBM (1, 2.5, 5, 10 mM), succinate (0.5, 1, 3, 5, 10 μg/mL) or sulfadiazine/(SD, 100 μg/mL, Bolida Technology Co., Ltd, Xuzhou, China) at a density of 2.5 × 10^4^/well in a 96-well microplate for 36 h. Then, the tachyzoites were collected and stained with trypan blue. The numbers of stained tachyzoites were counted using a microscope (OLYMPUS IX51). The inhibition rate of *T. gondii* tachyzoite = number of trypan blue stained tachyzoites/total tachyzoite numbers. Each group sets 6 duplicate wells.

## Statistical analysis

Data were analyzed using GraphPad Prism 8.0 and expressed as mean ± standard deviation (SEM). After the Shapiro-Wilk normality test was performed, the Student’s t-test was used to compare the two groups. Notably, Two-way ANOVA was used to analyze the effects of *T. gondii* infection, DBM treatment, and their interaction. If the main and interactive effects were defined, post hoc comparisons were performed using Tukey’s multiple comparisons to assess the statistical significance among groups. A *P*-value <0.05 was considered to indicate statistical significance.

## Results

### T. gondii chronic infection induces anxiety-like behaviors in mice

**Figure 1.**
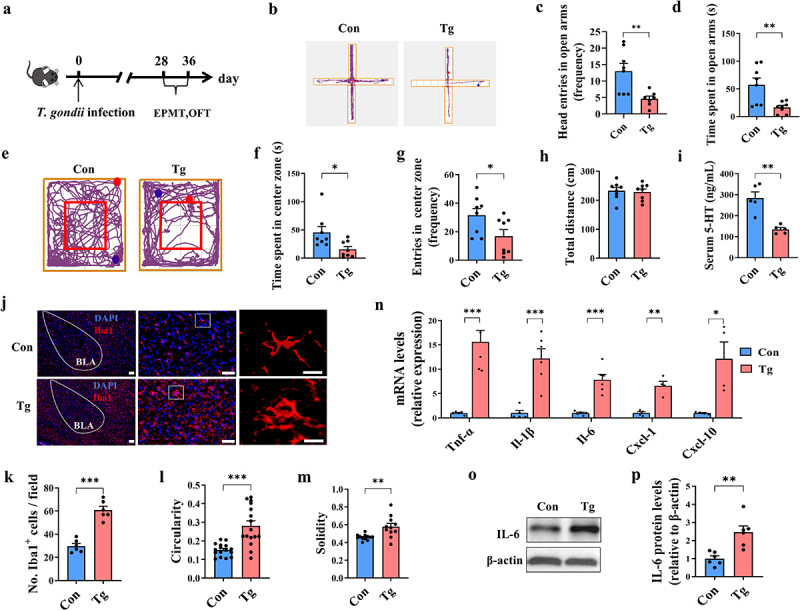
*T. gondii* chronic infection induces anxiety-like behaviors in mice. (a) schematic timeline for the model establishment of anxiety-like behaviors induced by *T. gondii* infection in mice. (b–d) EPMT test was performed to evaluate the anxiety-like behaviors of the mice (*n* = 8). (b) Representative movement traces of Con and Tg groups. (c) The frequency of head entries in open arms. (d) Time of head entries in open arms. (e–h) OFT test was performed to evaluate the anxiety-like behaviors of the mice (*n* = 8). (e) Representative movement traces of Con and Tg groups. (f) Time spent in center zone. (g) The frequency of head entries in center zone. (h) Total length of displacement distance. (i) Level of 5-HT in the serum (*n* = 5). (j) Immunofluorescence staining for iba1 in the amygdala (scale bar 200μm). The image was captured from the box (scale bar 50μm). (k) Quantification of Iba1^+^ microglia in the amygdala. Cell circularity (l) and solidity (m) were used to describe microglial morphology. Resting microglia show a high level of differentiation, whereas activated microglia show large cytostomes with no or very small bifurcations. Activated microglia possess a higher circularity and solidity. (n) mRNA expression of *Tnf-α*, *Il-1β*, *Il-6, Cxcl-1* and *Cxcl-10* in the amygdala (*n* = 4~6). (o) The representative bands of IL-6 in the amygdala. (p) The protein expression levels of IL-6 in the amygdala (*n* = 6). Con: control group; Tg: the mice were infected with *T. gondii* cysts. Values are presented as mean±SEM. *p < 0.05, **p < 0.01, ***p < 0.001.

The *T. gondii*-infected mouse model was established by inoculating intragastrically with *T. gondii* cysts, and the anxiety-like behaviors were evaluated by EPMT and OFT (Figure 1a). In the EPMT, the increase of anxiety was evidenced by the number of head-pokes and entries into the open arms. Compared with the Con group, the frequency of head entries in open arms of Tg-infected mice were significantly reduced, which was synchronized with the time spent in open arms (Figure 1b–d). In the OFT, the time spent in center zone in the Tg group was significantly lower than those in the Con group (Figure 1f). Moreover, the frequency of head entries in center zone in the Tg group was significantly lower (Figure 1g). In addition, Tg group mice had a lower desire to explore the middle zone (Figure 1e–g). However, the total length of displacement distance was similar between the two groups (Figure 1h), suggesting that the anxiety-like behaviors were not caused by affecting the motor ability of the mice. Furthermore, the level of 5-HT (a neurotransmitter closely related to anxiety) in the Con group was significantly higher than that in the Tg group (Figure 1i). Overall, these results suggest that *T. gondii* chronic infection induces anxiety-like behaviors in mice.

Next, we assessed the global expression pattern of genes associated with anxiety in the amygdala of mice post infection. Differentially expressed genes (DEGs) were identified after filtering the raw data according to *p* < 0.05 and |log_2_fold change| > 0.58 (Supplementary Figure S1A). There were 419 DEGs (including 364 upregulated genes and 55 downregulated genes) in the amygdala of Tg group compared with Con group (Supplementary Figure S1B). Notably, a large number of genes enriched in the neurotransmitter receptor were significantly regulated, including *Htr2c*, *Socs3* (Supplementary Figure S1C). For example, *Htr2c* (known as the 5-hydroxytryptamine receptor 2C) plays an important role in the 5-HT transport system, which is closely associated with anxiety and depression.^43^ Gene ontology (GO) analysis showed that the significantly enriched terms of these DEGs were associated with neurotransmission and cellular component (Supplementary Figure S1D,E). KEGG analysis showed significant enrichment of infection, neurotransmitter, immunity (Toxoplasmosis, Cytokine-cytokine receptor interaction, NF-kappa B signaling pathway, TNF signaling pathway, etc.) (Supplementary Figure S1F). Overall, these results suggest that *T. gondii* infection alters the expression profile of anxiety-associated transcripts in the amygdala of mice.

Neuroinflammation in the amygdala is an important neuropathological basis for anxiety.^23^ Here, we determined the profile of microglia in the basolateral amygdala (BLA) of infected mice (Figure 1j). Compared with the Con group, the number of Iba1^+^ cells (microglia marker) was significantly increased in the Tg group (*p* < 0.001, Figure 1k). Moreover, the majority of Iba1^+^ cells in the Tg group showed the morphology of activated microglia, which was evidenced by hypertrophic cell bodies, fewer branches, and increased circularity and solidity indices (Figure 1l,m). Correspondingly, the mRNA expression of pro-inflammatory cytokines (*Tnf-α*, *Il-1β* and *Il-6)* and chemokines (*Cxcl-1* and *Cxcl-10*) was significantly upregulated post *T. gondii* infection (all *p* < 0.05, Figure 1n). Accordingly, the *T. gondii* infection significantly increased the protein levels of *Il-6* in the amygdala compared to the Con group (Figure 1o,p). Overall, these results suggest that *T. gondii* chronic infection induces microglial activation and neuroinflammation in the amygdala of mice.

### T. gondii chronic infection induces the dysbiosis of gut microbiota and colonic inflammation in mice

**Figure 2.**
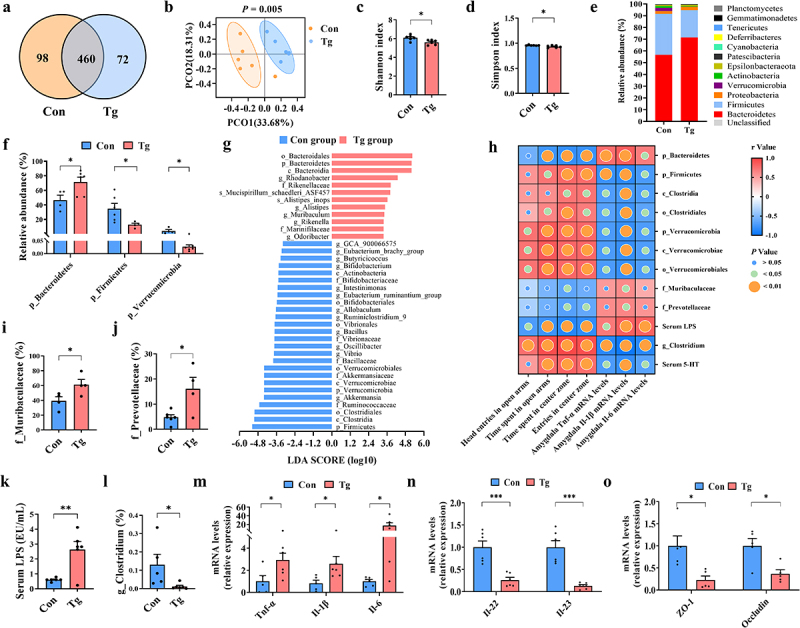
*T. gondii* chronic infection induces the dysbiosis of gut microbiota and colonic inflammation in mice. Fecal microbiota composition was analyzed by 16S rRNA gene sequencing (*n* = 6). (a) The number of shared and unique core OTUs between the Con and Tg groups. (b) Principal co-ordinate analysis of Bray distance (*p* value from Anosim analysis is shown). (c) Shannon index. (d) Simpson index. (e) Composition abundance of the bacterial phylum. (f) Comparison of the representative taxonomic abundance among Con and Tg groups. (g) Linear discriminant analysis (LDA) effect size (LEfSe) showing the most differentially significant abundant taxa enriched in microbiota. (h) Spearman’s correlation among the effects of *T. gondii* chronic infection on gut microbiota, neuroinflammation of the amygdala, and anxiety-like behaviors. (i–j) Relative abundance of LPS-producing bacteria in the Con and Tg groups. (k) The level of LPS in the serum (*n* = 6). (l) Relative abundance of 5-HT-producing bacteria. (m) mRNA expression of *Tnf-α*, *Il-1β*, *Il-6* in the colon (*n* = 4~6). (n) mRNA expression of *Il-22*, *Il-23* in the colon (*n* = 4~6). (o) mRNA expression of ZO-1, occludin in the colon (*n* = 4~6). Values are presented as mean±SEM. *p < 0.05, **p < 0.01, ***p < 0.001.

Previous studies indicated that gut microbiota is closely associated with anxiety.^19^ Here, we characterized the profile of gut microbiota post *T. gondii* chronic infection. The average operational taxonomic units (OTUs) that overlap and are unique between the Con and Tg groups were presented by Venn diagrams, with 460 OTUs shared between the two groups, 98 unique OTUs observed in the Con group, and 72 unique OTUs in the Tg group (Figure 2a). Principal component analysis (PCoA) reflected a significant separation between two groups (Anosim, *p* = 0.005; Figure 2b). Moreover, in compared to the Con group, the Shannon and Simpson indices were lower in the Tg group, indicating a significant decrease in species diversity post *T. gondii* infection (Figure 2c,d). In addition, at the phylum level, *T. gondii* infection markedly reduced the relative abundance of *Firmicutes* and *Verrucomicrobia*, and increased the relative abundance of *Bacteroidetes* (Figure 2e,f).

Linear discriminant analysis (LDA) effect size (LEfSe) revealed differential enrichment (LDA score > 3) among bacteria at various taxonomic levels, including the representative bacteria belonging to the *Bacteroidetes*, *Firmicutes*, and *Verrucomicrobia* at the phylum level, in which, *Bacteroidia*, *Clostridia*, and *Verrucomicrobiae* at the class level; and *Bacteroidales*, *Clostridiales*, and *Verrucomicrobiales* at the order level were differential enrichment (Figure 2g). Interestingly, the abundance of LPS-producing bacteria (*Muribaculaceae* and *Prevotellaceae*), was significantly increased in the Tg group (Figure 2i,j), which contributed to the increased LPS levels in the serum of infected mice (Figure 2k). Moreover, the infection increased the abundance of mucus layer degradation associated bacteria (*Prevotellaceae_UCG-001* and *Bacteroides*) (Supplementary Figure S2A,B); however, decreased the abundance of *Clostridium* (one of 5-HT-producing bacteria) (Figure 2l).

Spearman’s correlation analysis showed that the anxiety-like behaviors and neuroinflammation were positively correlated with the abundance of *Bacteroidetes*, LPS-producing bacteria and the levels of LPS, while negatively correlated with the abundance of bacteria belonging to *Firmicutes* and *Verrucomicrobia* phylum, 5-HT-producing bacteria and the levels of 5-HT (Figure 2h). Overall, these results suggest that *T. gondii* chronic infection induces gut microbiota dysbiosis, which may be associated with anxiety-like behaviors and neuroinflammation.

The intestinal immune cells and their derived cytokines can influence the composition of the intestinal microbiota.^44^ We found that the mRNA expression of pro-inflammatory cytokines *Tnf-α*, *Il-1β* and *Il-6* in the colon was significantly upregulated in the Tg group (Figure 2m). On the contrary, the mRNA expression of *Il-22 and Il-23*, two cytokines that maintain the homeostasis of gut microbiota,^45^ was obviously downregulated post infection (Figure 2n). In addition, we found the decreased mRNA expression of the tight junction-associated genes (ZO-1 and occludin) in the colon of Tg group (Figure 2o), which was consistent to elevated levels of FITC-dextran in the plasma and the decreased number of ZO-1^+^ cells in the colon of infected mice (Supplementary Figure S2C–E). Taken together, these results suggest that *T. gondii* chronic infection compromises the gut barrier and causes the immune dyshomeostasis in the colon of mice.

### *Elimination of gut microbiota ameliorates T. gondii*-*induced-anxiety-like behaviors*

**Figure 3.**
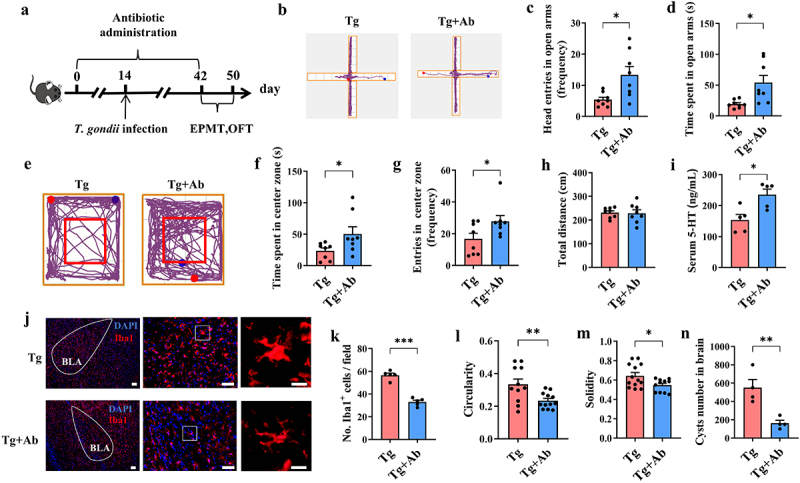
Elimination of gut microbiota ameliorates *T. gondii*-induced-anxiety-like behaviors. (a) schematic timeline for the model establishment of the Tg+Ab group. (b–d) EPMT test was performed to evaluate the anxiety-like behaviors of the mice (*n* = 8). (b) Representative movement traces of Tg and Tg+Ab groups. (c) The frequency of head entries in open arms. (d) Time of head entries in open arms. (e–h) OFT test was performed to evaluate the anxiety-like behaviors of the mice (*n* = 8). (e) Representative movement traces of Tg and Tg+Ab groups. (f) Time spent in center zone. (g) The frequency of head entries in center zone. (h) Total length of displacement distance. (i) Level of 5-HT in the serum (*n* = 5). (j) Immunofluorescence staining for iba1 in the amygdala (scale bar 200μm). The image was captured from the box (scale bar 50μm). (k) Quantification of Iba1^+^ microglia in the amygdala. Cell circularity (l) and solidity (m) were used to express microglial morphology. (n) Cyst number in the brain (*n* = 4). Tg: *T. gondii*-infected group; Tg+Ab: the mice were infected with *T. gondii* cysts and treated with antibiotics until sacrificed. *n* = 8. Values are presented as mean±SEM. *p < 0.05, **p < 0.01, ***p < 0.001.

To identify the role of gut microbiota in *T. gondii*-induced anxiety, broad-spectrum antibiotic (Ab) treatment was used to eliminate gut microbiota in *T. gondii*-infected mice (Figure 3a). Ab cocktail treatment just slightly decreased the body weight of control mice (Supplementary Figure S3A), indicating that mice on Ab water were equally healthy to non-treated controls. In the EPMT, Ab treated Tg-infected mice showed increased frequency in open arm and elevated time spent in the open arm compared to the Tg group (Figure 3b–d). In the OFT, Ab treatment increased the time mice spent in the central zone and the frequency of head entries in center zone (Figure 3e–g). Notably, the total distance moved in the Tg+Ab group was similar to that of the Tg group (Figure 3h). Moreover, Ab treatment also elevated the levels of 5-HT in the serum of Tg group (Figure 3i). In addition, Ab treatment eliminated microgliosis as well as neuroinflammation in the amygdala (Figure 3j–m). Thus, antibiotic treatment of gut microbiota alleviates *T. gondii*-induced anxiety and neuroinflammation in mice.

Previous studies have reported that the cyst burden of *T. gondii* is closely associated with behavior alteration and neuroinflammation.^9,10^ Here, we showed that the cyst number was significantly decreased in the brains of the Tg+Ab group compared with the Tg+Veh group (Figure 3n). To verify whether Ab has a direct effect on *T. gondii* survival, the tachyzoites of *T. gondii* were cultured in the presence of Ab cocktail with different concentrations. Similar to SD (the anti-*T. gondii* drug used in clinic, 100 μg/mL) treatment, 200 μg/mL Ab cocktail including neomycin (61.54 μg/mL), ampicillin (61.54 μg/mL), metronidazole (61.54 μg/mL), and vancomycin (15.38 μg/mL), significantly decreased the number of live tachyzoites. However, 20, 50, and 100 μg/mL Ab cocktails could not affect the survival of tachyzoites (Supplementary Figure S4A). Thus, it is possible that a high concentration of Ab treatment may directly decrease the cyst burden.

### *Gut microbiota dysbiosis contributes to T. gondii*-*induced anxiety-like behaviors and gut barrier impairment in mice*

**Figure 4.**
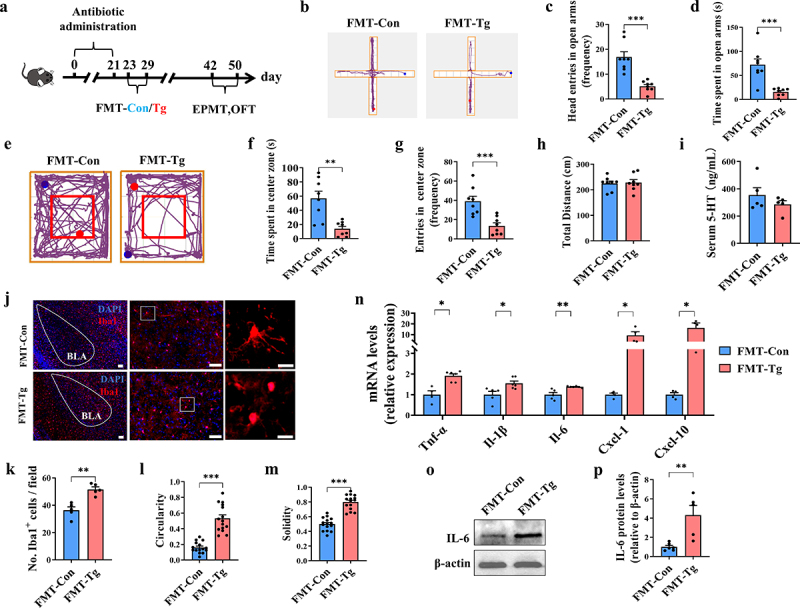
Gut microbiota dysbiosis contributes to *T. gondii*-induced anxiety-like behaviors and gut barrier impairment in mice. (a) schematic timeline for the model establishment of the FMT-Con and the FMT-Tg groups. (b–d) EPMT test was performed to evaluate the anxiety-like behaviors of the mice (*n* = 8). (b) Representative movement traces of the FMT-Con and FMT-Tg groups. (c) The frequency of head entries in open arms. (d) Time of head entries in open arms. (e–h) OFT test was performed to evaluate the anxiety-like behaviors of the mice (*n* = 8). (e) Representative movement traces of the FMT-Con and FMT-Tg groups. (f) Time spent in center zone. (g) The frequency of head entries in center zone. (h) Total length of displacement distance. (i) Level of 5-HT in the serum (*n* = 5). (j) Immunofluorescence staining for iba1 in the amygdala (scale bar 200μm). The image was captured from the box (scale bar 50μm). (k) Quantification of Iba1^+^ microglia in the amygdala. Cell circularity (l) and solidity (m) were used to express microglial morphology. (n) mRNA expression of *Tnf-α*, *Il-1β*, *Il-6*, *Cxcl-1* and *Cxcl-10* in the amygdala (*n* = 4~6). (o) The representative bands of IL-6 in the amygdala. (p) The protein expression levels of IL-6 in the amygdala (*n* = 6). FMT-Con: the mice were given antibiotics for three weeks first and then transplanted fecal microbiota from Con mice; FMT-Tg: the mice were given antibiotics for three weeks first and then transplanted fecal microbiota from Tg-infected mice. Values are presented as mean±SEM. *p < 0.05, **p < 0.01, ***p < 0.001.

To further uncover the causal relationship between gut microbiota dysbiosis and anxiety-like behaviors, we transferred the fecal microbiota from Tg and Con mice to the C57BL/6J mice pretreated with Ab cocktail treatment (Figure 4a). Compared to control mice, Ab cocktail treated mice showed an approximately 20-fold reduction in fecal bacterial load (Supplementary Figure S3B). Notably, similar to the microbiota pattern of *T. gondii*-infected mice, the recipient mice receiving the gut microbiota from *T. gondii*-infected mice (FMT-Tg) had a decrease in the abundance of *Firmicutes* and *Verrucomicrobia* phylum, and the increase in the abundance of Bacteroidetes phylum (Supplementary Figure S5A-D), indicating the successful transplantation of donors’ gut microbiota to the recipient mice.

In the EPMT, FMT-Tg group showed a significant reduction in the frequency of head entries in open arms and the time spent in open arms (Figure 4b–d). In the OFT, the time spent in the central area in the FMT-Tg group was significantly lower than that in the FMT-Con group (the recipient mice receiving the gut microbiota from the control mice). Meanwhile, the frequency of head entries into the central area was also significantly lower in the FMT-Tg group (Figure 4e–g). There was no difference in the total time spent exploring objects between the two groups during the testing phase, indicating that the motor ability of the mice was not affected (Figure 4h). Moreover, the serum level of 5-HT in the FMT-Tg group was slightly lower than that in the FMT-Con group (Figure 4i). In addition, the FMT-Tg group exhibited microglia activation and neuroinflammation (Figure 4j–m), evidenced by the increased number of microglia, upregulated mRNA level of pro-inflammatory cytokines, chemokines and protein level of IL-6 in the amygdala of FMT-Tg mice (Figure 4n–p). However, we did not find the cysts of *T. gondii* in the brains of FMT-Tg group.

Furthermore, we evaluated the effects of gut microbiota transplantation on the colonic pro-inflammatory response and barrier function in the recipient mice. In comparison to the FMT-Con group, the FMT-Tg group showed the upregulated expression of pro-inflammatory cytokines (*Tnf-α*, *Il-1β* and *Il-6*) and the decreased expression of immune homeostasis-related cytokines (*Il-22* and *Il-23*) in the colon (Supplementary Figure S6A,B). Moreover, the FMT-Tg downregulated the mRNA expression of tight junction proteins (ZO-1 and occludin) in the recipient mice (Supplementary Figure S6C). In addition, fewer ZO-1^+^ cells were observed in the colon of the FMT-Tg group. Correspondingly, compared to the FMT-Con group, both levels of LPS and FITC-dextran were significantly higher in the FMT-Tg group (Supplementary Figure S6D–G). Overall, these results indicate that the gut microbiota from the *T. gondii* infected mice induces the disorder of gut-brain axis and anxiety in the recipient mice, which is similar to the phenotype observed in *T. gondii*-infected mice, highlighting the critical role of gut microbiota in mediating the anxiety in *T. gondii*-infected mice.

### *Transplantation of the fecal microbiota from T. gondii*-*infected mice alters the transcriptomic profile associated with anxiety in the amygdala of mice*

**Figure 5.**
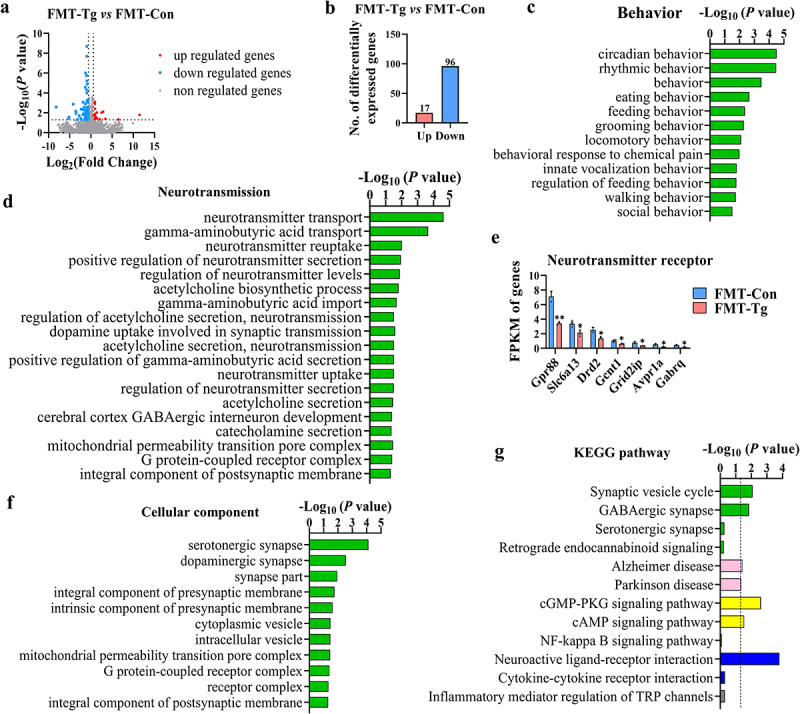
Transplantation of the fecal microbiota from *T. gondii* infected mice alters the transcriptomic profile associated with anxiety in the amygdala of mice. The transcriptomic profile of the amygdala was determined by Transcriptome sequencing (*n* = 3). (a) The volcano plot shows the distributions of differentially expressed genes (DEGs) between the FMT-Con and FMT-Tg mice. (b) The number of upregulated and downregulated DEGs. (c) The biological processes associated with behavior are significantly enriched in the FMT-Tg group. (d) The biological processes associated with neurotransmission are significantly enriched in the FMT-Tg group. (e) Normalized expression of selected genes regulating neurotransmitter receptor. FPKM (fragment per kilobase of transcript per million mapped reads) value was calculated to quantify gene’s expression abundance and variations. (f) The biological processes associated with cellular component are significantly enriched in the FMT-Tg group. (g) The enriched KEGG pathways related to neurotransmitter, immunity and metabolism in the FMT-Tg group. Columns with different colors represent different classifications in level 2. The dotted line in the figure represents *p* = 0.05. Values are presented as mean±SEM. *p < 0.05, **p < 0.01.

We next assessed the global expression pattern of genes associated with anxiety in the amygdala of FMT recipient mice. Differentially expressed genes (DEGs) were identified after filtering the raw data according to *p* < 0.05 and |log_2_fold change| > 0.58 (Figure 5a). There were 113 DEGs (including 17 upregulated genes and 96 downregulated genes) in the amygdala of FMT-Tg group compared with FMT-Con group (Figure 5b). Gene ontology (GO) analysis showed that the significantly enriched terms of these DEGs were associated with behavior and neurotransmission (Figure 5c,d). Notably, a large number of genes enriched in the neurotransmitter receptor were significantly down-regulated, including *GPr88*, *Slc6a13*, *Drd2* (Figure 5e). For example, *Slc6a13* (known as the plasma membrane GABA transporter GAT2) plays an important role in the γ-aminobutyric acid (GABA) transport system, which is closely associated with anxiety.^46^

Moreover, the cellular component of DEGs such as serotonergic synapse and dopaminergic synapse were also significantly enriched post infection (Figure 5f). KEGG analysis showed significant enrichment of neurotransmitter, immunity, and metabolism-related pathways (GABAergic synapse, Serotonergic synapse, NF-kappa B signaling pathway, cGMP-PKG signaling pathway, etc.) (Figure 5g). Neurotransmitters have long been regarded as central to the regulation of anxiety^47,48^ To comprehensively assess the neurotransmitter function, we performed a Gene Set Enrichment Analysis (GSEA) to identify enriched gene sets for specific GO or KEGG pathways. We found significant differences in the gene set “dopaminergic synapse” (Supplementary Figure S7). It is reported that higher levels of anxiety are associated with reduced dopamine neurotransmission.^49^ Overall, these results suggest that transplantation of fecal microbiota from *T. gondii*-infected mice alters the expression profile of anxiety-associated transcripts in the amygdala of mice, which further explains the anxiety-like behaviors induced by the gut microbiota from *T. gondii*-infected mice.

### T. gondii chronic infection decreases the abundance of succinate-producing bacteria and succinate production in the feces of mice

**Figure 6.**
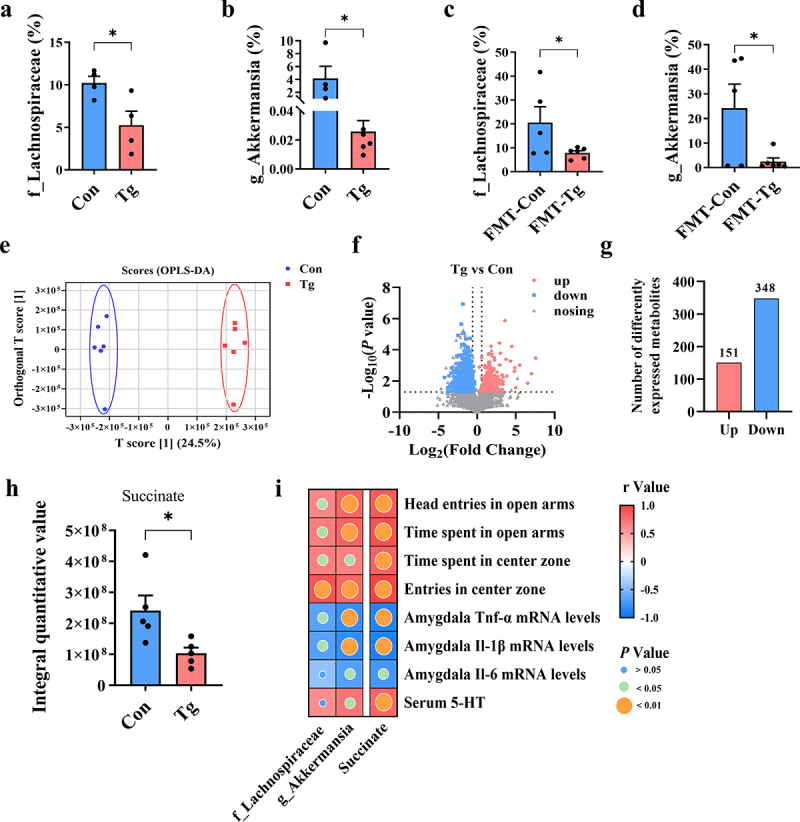
*T.* *gondii* chronic infection decreases the abundance of succinate-producing bacteria and succinate production in the feces of mice.

We were interested in which gut microbiota or its metabolites trigger the anxiety-like behaviors in the infected mice. A previous study has suggested that succinate-producing bacteria may be closely related to anxiety.^50^ Here, we found that the abundance of two succinate-producing bacteria (*Lachnospiraceae* and *Akkermansia)*, was significantly decreased in the Tg group (Figure 6a,b). Interestingly, a similar abundance alteration of the two bacteria occurred in the FMT-Tg group (Figure 6c,d).

(a-b) Relative abundance of succinate-producing bacteria. (c-d) Relative abundance of succinate-producing bacteria. (e) Score chart of OPLS-DA. (f) Volcano map of differential metabolite. (g) The number of differential metabolites expressed by bar-chart. (h) Integral quantitative value of succinate in the feces. (i) The association between intestinal succinate and neuroinflammation and anxiety-like behaviors. *n* = 6. Values are presented as mean ± SEM. *p < 0.05.

To further investigate the potential mechanism by which the gut microbiota contributes to anxiety, we detected the profiles of differential metabolites in the feces of donor and recipient mice. Score plots of orthogonal partial least-squares discriminant analysis (OPLS-DA) distinctly separated the Tg group from the Con group (Figure 6e,f). There were 151 upregulated metabolites and 348 downregulated metabolites between the Con and Tg groups (Figure 6g). Interestingly, the levels of succinate were significantly decreased in the feces of Tg group, which is consistent with changes in succinate-producing bacteria (Figure 6h). Correspondingly, OPLS-DA distinctly separated the FMT-Tg group from the FMT-Con group (Supplementary Figure S8A). There were 56 upregulated metabolites and 45 downregulated metabolites between the FMT-Con and FMT-Tg groups (Supplementary Figure S8B–D). Notably, compared to the FMT-Con group, the integral quantitative value of methylsuccinate, a kind of succinate derivative, was reduced significantly in the FMT-Tg group (Supplementary Figure S8E). Thus, it is proposed that a decreased abundance of succinate-producing bacteria contributes to the lower levels of succinate in the feces of Tg and FMT-Tg mice. On the contrary, succinate levels were slightly increased in the serum of the Tg group (Supplementary Figure S9A). Furthermore, Spearman’s correlation analysis showed that both the abundance of *Lachnospiraceae* and *Akkermansia*, and succinate production in the feces were negatively correlated with anxiety-like behaviors and neuroinflammation, but positively correlated with the levels of 5-HT (Figure 6i, Supplementary Figure S8F). Thus, we speculated that the decreased succinate produced by gut microbiota may be associated with anxiety-like behaviors.

### Diethyl butylmalonate ameliorates the anxiety-like behaviors and the adverse effects on the gut-brain axis induced by T. gondii chronic infection

**Figure 7.**
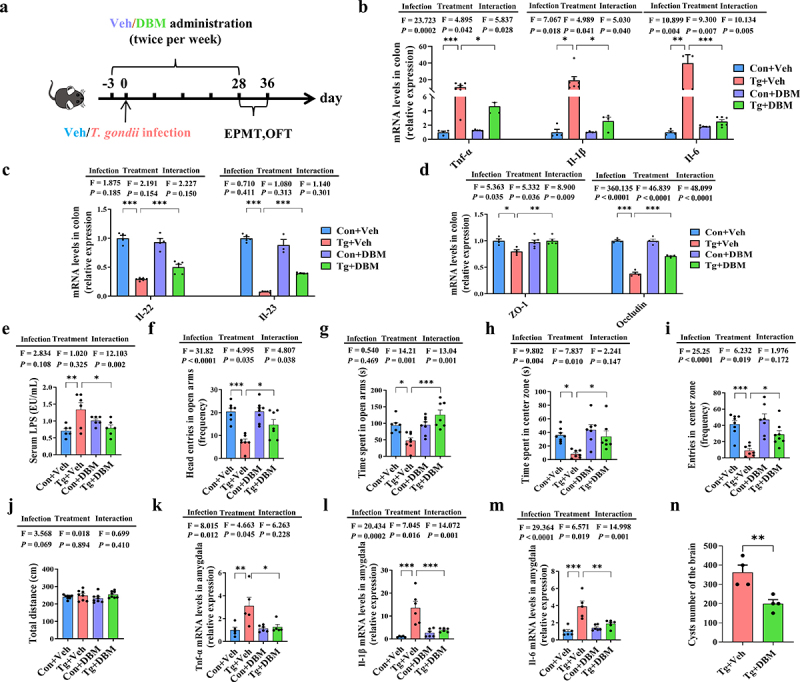
Diethyl butylmalonate ameliorates the anxiety-like behaviors and the adverse effects on the gut-brain axis induced by *T. gondii* chronic infection. (a) schematic timeline for model establishment of Con+Veh, Tg+Veh, Con+DBM and Tg+DBM groups (*n* = 8). (b) mRNA expression of *Tnf-α*, *Il-1β*, *Il-6* in the colon (*n* = 4~6). (c) mRNA expression of *Il-22*, *IL-23* in the colon (*n* = 4~6). (d) mRNA expression of ZO-1, occludin in the colon (*n* = 4~6). (e) Level of LPS in the serum (*n* = 6). (f–g) EPMT test was performed to evaluate the anxiety-like behaviors of the mice (*n* = 8). (f) The frequency of head entries in open arms. (g) Time spent in open arms. (h–j) OFT test was performed to evaluate the anxiety-like behaviors of the mice (*n* = 8). (h) Time spent in center zone. (i) The frequency of head entries in center zone. (j) Total length of displacement distance. (k–m) mRNA expression of *Tnf-α*, *Il-1β*, *Il-6* in the amygdala (*n* = 4~6). (n) Cyst number in the brain (*n* = 4). Con+Veh: control group and treated with PBS; Tg+Veh: the mice were infected with *T. gondii* cysts and treated with PBS; Con+DBM: the mice were treated with DBM; Tg+DBM: the mice were infected with *T. gondii* cysts and treated with DBM. Values are presented as mean±SEM. *p < 0.05, **p < 0.01, ***p < 0.001.

In order to investigate the role of succinate in *T. gondii*-mediated anxiety-like behaviors, we pre-treated the Tg-infected mice with the DBM (an inhibitor of the mitochondrial succinate transporter that can cause succinate accumulation) (Figure 7a). DBM supplementation significantly increased the serum levels of succinate in the Con+DBM and Tg+DBM groups (Supplementary Figure S9A). We observed the downregulated mRNA expression of pro-inflammatory cytokines (*Tnf-α*, *Il-1β*, *Il-6*) in the colon of the Tg group after DBM administration, suggesting that DBM can alleviate the intestinal inflammation (Figure 7b). Moreover, DBM elevated the mRNA expression of the immune homeostasis related cytokines (*Il-22* and *Il-23*) and tight junction proteins (ZO-1 and occludin) in the colon of the Tg group (Figure 7c,d), which was accompanied by the decreased levels of LPS in the serum of those mice (Figure 7e). These results indicate that DBM can repair the impairment of the gut barrier.

Furthermore, we evaluated the effect of DBM administration on the anxiety-like behaviors induced by *T. gondii* chronic infection. In the OFT and EPMT, DBM treatment significantly increased the desire of infected mice to explore the open arm or center area (all *p* < 0.05, Figure 7f–j, Supplementary Figure S9B, C). DBM administration also elevated 5-HT levels in the serum of infected mice (Supplementary Figure S9D). Moreover, DBM administration alleviated microglia gliosis and activation in the BLA of Tg mice (Supplementary Figure S9E–H). Correspondingly, DBM administration downregulated the expression of *Tnf-α*, *Il-1β* and *Il-6* in the amygdala of the Tg group (Figure 7k–m). In addition, the cyst number was significantly decreased in the Tg+DBM group compared with the Tg+Veh group (*p* < 0.05, Figure 7n). It should be pointed out that DBM with different concentrations did not directly affect the survival of *T. gondii* tachyzoites *in vitro* (Supplementary Figure S4B). Moreover, succinate with the concentration comparable to serum level in mice (Supplementary Figure S9A), could not inhibit the growth of *T. gondii* (Supplementary Figure S4C).

## Discussion

**Figure 8.**
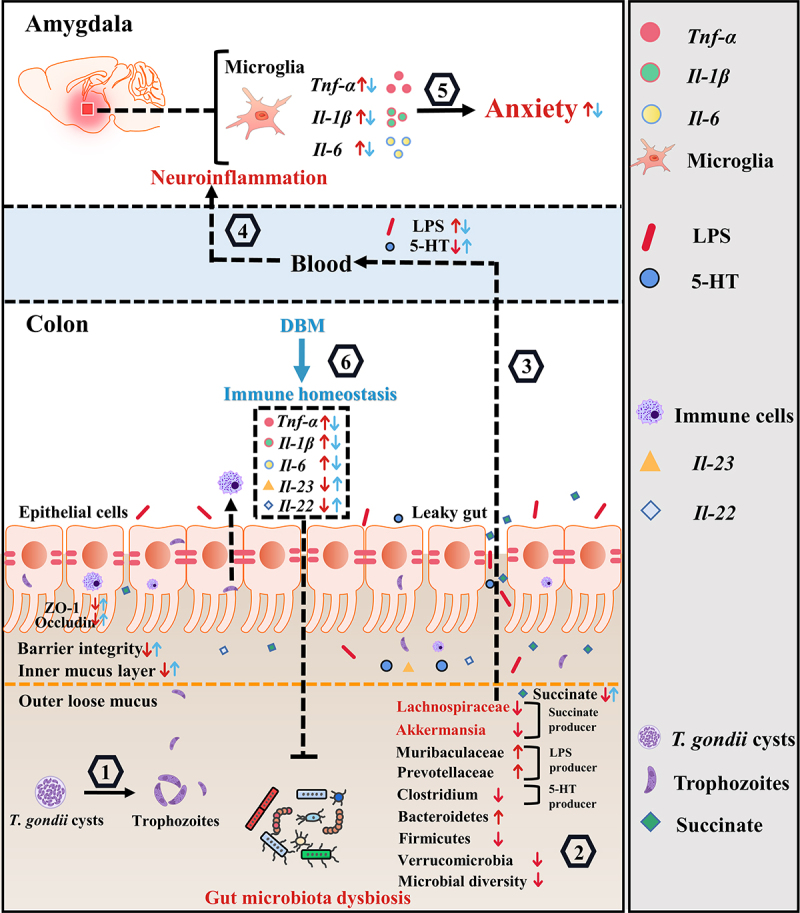
Schematic strategy for DBM’s role in improving anxiety-like behaviors induced by *T. gondii* chronic infection *via* the gut-brain axis. *T*. *gondii* chronic infection induces colonic inflammation, impairment of gut barrier integrity, and gut microbiota dysbiosis, characterized by the decreased abundance of succinate- and 5-HT-producing bacteria and the increased abundance of LPS-producing bacteria (1-2). These events lead to elevated LPS and deceased 5-HT levels in the serum of infected mice (3). LPS can trigger peripheral inflammation, allowing pro-inflammatory cytokines to enter the amygdala to activate microglia (4), and consequently, causing anxiety (5). However, DBM administration can resist these adverse effects *via* partially alleviating colonic inflammation and barrier integrity impairment (6). Red arrows represent altered indices induced by chronic infection of *T. gondii*, while blue arrows show the protective effects of DBM.

In the present study, we uncovered that gut microbiota plays an essential role in *T. gondii*-associated anxiety and the disorder of gut-brain axis (Figure 8). We showed that the chronic infection of *T. gondii* induced the anxiety-like behaviors, accompanied by decreased 5-HT and upregulated neuroinflammation in the amygdala. This is consistent with the results in previous studies.^7,10^ Moreover, we reported that *T. gondii* infection impaired the integrity of gut barrier. In addition, we showed that *T. gondii* infection induced the dysbiosis of gut microbiota, which was characterized by the decreased abundance of succinate-producing bacteria and the lower succinate production in the colonic feces. Interestingly, the anxiety-like behaviors and the disorder of gut-brain axis induced by *T. gondii* infection were observed in the FMT-Tg mice. The broad-spectrum Ab cocktail intervention attenuated these mentioned adverse effects. These findings highlight the important role of the gut microbiota in the anxiety-like behaviors. Notably, we showed that DBM could significantly ameliorate *T. gondii*-induced anxiety and the impairment of the gut barrier. Taken together, these findings provide a novel insight into the pathogenesis and therapeutic strategy of *T. gondii-*related mental disorder in the view of the microbiota-gut-brain axis (Figure 8).

Accumulated studies have shown that chronic infection of *T. gondii* is associated with neuropsychiatric disorders, including anxiety, dementia, and personality changes.^51–53^ For example, in the investigations on human subjects, those with serological evidence of *T. gondii* exposure showed positive correlations between IgG levels and anxiety.^11,12^ Moreover, various studies have reported that *T. gondii* can induce anxiety-like behaviors in mice.^7,9,10^ However, in addition to increases, decreases and no effect on anxiety-like behavior have also been reported in *T. gondii*-infected rodents.^54^ Based on the literature, the inconsistencies in behavioral changes may be attributed to differences in *T. gondii* strains, number of strains, infection mode, host species, and methods of measuring behavior.^55,56^ Here, we showed that *T. gondii* infection induces anxiety-like behaviors in mice. This behavior alteration was not caused by affecting the motor ability of the mice, because there was no significant difference in total distance of test phases between the Con and Tg groups. 5-HT is a neurotransmitter that acts on the central nervous system (CNS), blood factors and neurohormones, and its concentration is strongly associated with anxiety.^57^ We showed a decreased level of 5-HT in the serum of *T. gondii-*infected mice.

Furthermore, neuroinflammation in the amygdala is regarded as the important neuropathological basis for anxiety.^23^ Microglia play a key role in maintaining homeostasis in the brain.^58^ We observed the increased number of microglia in the BLA of amygdala of mice post infection. Since microglia quantification may be not a typical feature of neuroinflammation,^59^ we further evaluated the morphological changes of microglia. We found hypertrophic cell bodies, fewer branches and increased circularity and solidity index of Iba1^+^ cells post infection, indicating the activation of microglia.^60^ Correspondingly, we showed that the infection significantly elevated the mRNA expression of *Il-6*, *Il-1β*, *Tnf-α, Cxcl-1* and *Cxcl-10* in the amygdala of mice. Notably, these pro-inflammatory cytokines and chemokines have been reported to participate in the induction of anxiety in mice.^41,61^ Thus, chronic infection of *T. gondii* contributes to neuroinflammation in the amygdala. It should be pointed out that other brain regions including the hippocampus, are also involved in anxiety.^62,63^ We found the elevated expression of pro-inflammatory cytokines in the hippocampus, which was similar to the phenotype observed in the amygdala (Supplementary Figure S10a, b). Future work should integrate multiple regions to explore the underlying mechanism of how *T. gondii* induces anxiety.

Mental disorders and neuroinflammation can be regulated by the gut microbiota *via* the gut-brain axis.^64^ Here, we found the reduced diversity indices (Shannon index and Simpson index) of gut microbiota, increased abundance of phylum *Bacteroidetes*, and decreased abundance of phylum *Firmicutes* and *Verrucomicrobia* in *T. gondii*-infected mice. The LDA analysis further showed that the infection decreased not only the *Firmicutes* and *Verrucomicrobia* at phylum but also their lower taxa, such as *Clostridia* and *Verrucomicrobiae* at class, *Clostridiales* and *Verrucomicrobiales* at order, *Lachnospiraceae* at family, *Akkermansia* at genus. Previous studies have shown that several bacteria (e.g., *Clostridium*), can synthesize 5-HT.^65–67^ Here, we observed the decreased abundance of *Clostridium* in *T. gondii*-infected mice, which is consistent with the lower levels of 5-HT in the serum of infected mice. Moreover, we found an increased abundance of LPS-producing bacteria (e.g., *Muribaculaceae* and *Prevotellaceae*)^68,69^ and elevated levels of serum LPS in the mice post infection. A recent study showed that LPS can damage blood-brain barrier integrity *in vivo*.^22^ Also, LPS is reported to upregulate the expression of TNF-α, IL-1β *via* activating microglia.^70,71^ Notably, these pro-inflammatory cytokines have been reported to induce anxiety-like behaviors.^16,17^ Thus, we proposed that disturbance of gut microbiota may decrease 5-HT levels and induce neuroinflammation in the amygdala, which jointly contribute to anxiety behaviors induced by *T. gondii* infection.

Previous studies have documented that the dysbiosis of gut microbiota can be induced by *T. gondii* infection. Moreover, microbial spectrums were more disordered in the chronic period compared with that in the acute period.^30–32^ However, the exact role of gut microbiota in *T. gondii*-associated anxiety has not been established. This study confirmed that the disordered gut microbiota is a vital factor for *T. gondii*-associated anxiety. Ab treatment can be used to explore the effects of gut microbiota on physiology and disease in mice.^72^ Here, we found that long-term administration of Ab cocktails attenuated the anxiety-like behaviors and neuroinflammation induced by *T. gondii* infection, without affecting the weight of these mice. Notably, the effects of *T. gondii* infection on anxiety and neuroinflammation were observed in the FMT-Tg mice, highlighting the critical role of the gut microbiome in mediating the anxiety. In addition, transcriptomic analysis supported that the microbiota from infected mice induced anxiety, evidenced by the enriched GO terms associated with behavior, neurotransmission, neuroinflammation, GABAergic synapse, Serotonergic synapse, and Dopaminergic synapse in the amygdala of the recipient mice. Previous studies also reported that fecal microbiota transplantation from mice with chronic unpredictable mild stress or patients with irritable bowel syndrome can promote anxiety-like behaviors in recipient mice.^73,74^ Collectively, these findings, including ours, indicate that gut microbiota dysbiosis plays a vital role in anxiety.

The crosstalk between gut microbiota and immunity is essential for gut homeostasis,^75,76^ and consequently regulating anxiety. *Prevotellaceae_UCG-001* and *Bacteroides* are reported to increase epithelial permeability *via* producing mucin-degrading enzyme;^77,78^ while *Muribaculaceae* and *Prevotellaceae* are LPS-producing bacteria.^68,69^ Here, we found that the abundance of the 4 bacteria was significantly elevated after *T. gondii* infection. Correspondingly, we found that the infection impaired the colonic barrier integrity, evidenced by the fewer ZO-1^+^ cells and the downregulated mRNA expression of tight function proteins associated genes (ZO-1 and occludin) in the colon,^79^ and the elevated levels of LPS and FITC-dextran. It is reported that intestinal barrier dysfunction may facilitate the entry of LPS into the blood circulation.^80^ Moreover, we showed that *T. gondii* infection upregulated the mRNA expression of pro-inflammatory cytokines (*Il-6*, *Il-1β* and *Tnf-α*) in the colon of mice. Interestingly, we observed that *T. gondii*-infected mice had lower mRNA expression of immune homeostasis-associated cytokines (*Il-22* and *Il-23*). Although IL-23 is known as a pro-inflammatory cytokine,^81^ it can activate Th17 to produce IL-22.^82^ It is reported that IL-22 can enhance the epithelial cell barrier by increasing the expression of Reg3γ, thereby reducing the invasion of microbiota.^45,83^ Notably, a similar expression pattern of these cytokines was also observed in FMT-Tg mice. Therefore, these results indicate that *T. gondii*-induced gut microbiota shift contributes to the loss of immune homeostasis in the colon of mice, which might further exacerbate the dysbiosis of gut microbiota. Furthermore, it has been reported that *T. gondii* infection activates gut innate and adaptive immune cell. For example, *T. gondii* preferentially infects infiltrating neutrophils to disseminate into other host tissues.^84^ Activated neutrophils can secrete cytokines important for type I immunity including IL-12 and TNF-α to kill extracellular tachyzoites.^85^ Moreover, CD8^+^T cells are reported to kill *T. gondii*-infected epithelial cells and contribute to type I immunity.^86^ Thus, a complex interplay among *T. gondii*, immune cells and gut microbiota may be jointly attributed to the disorder of gut function.

Furthermore, compared to the Con and FMT-Con group, the abundance of succinate-producing bacteria (*Lachnospiraceae* and *Akkermansia*),^24,25^ and succinate levels, were both decreased in the feces of Tg and FMT-Tg mice. It is reported that increased abundance of *Lachnospiraceae* helps to alleviate the depression-like behaviors induced by chronic unpredictable mild stress (CUMS).^26^ Moreover, a lower abundance of *Akkermansia* is reported in the social defeat mice with anxiety- and depressive-like behaviors.^27^ Notably, succinate is reported to influence the levels of GABA, which plays an important role in anxiety.^87^ Administration of desvenlafaxine succinate is reported to relieve anxiety symptoms associated with major depressive disorder in clinical trials.^88^ These findings attracted us to investigate the effect of succinate supplementation on anxiety in *T. gondii* infected mice. DBM is an inhibitor of the mitochondrial succinate transporter that causes endogenous succinate accumulation.^33,34^ Here, we reported that DBM supplementation can prevent *T. gondii*-induced anxiety. Moreover, DBM administration alleviated microglia gliosis and activation, downregulated the expression of *Tnf-α*, *Il-1β* and *Il-6* in the amygdala of the Tg mice. Meanwhile, DBM supplementation decreased the cyst number in the brains of infected mice. A previous study has reported that *T. gondii* cysts can induce neuroinflammation.^89^ It is therefore speculated that the decreased expression of inflammatory cytokines may result from the decreased cyst burden. However, both DBM and succinate could not inhibit the growth rate of *T. gondii in vitro*. Thus, the causal relationship between the decreased neuroinflammation and cyst burden needs to be further investigated in future.

In a recent study, intestinal succinate is reported to act as an important microbial product for the beneficial metabolic effects of dietary fiber.^90^ Notably, succinate is a classic intermediate for propionate synthesis.^91^ Here, in consistent with the decreased succinate production, we observed less propionate levels in the feces of infected mice (Supplementary Figure S11). Accumulated evidence has supported that propionate can maintain intestinal barrier integrity and regulate colonic inflammation.^92,93^ Interestingly, administration of propionate has been reported to improve stress response, anxiety, and depression.^94^ Propionate is also reported to ameliorate diabetic-induced depression-like behavior, spatial learning, and memory deficits.^95^ Moreover, orally administered propionate successfully rescued motor deficits and dopaminergic neuronal loss in a mouse model of Parkinson’s disease.^96^ Thus, it is speculated that *T. gondii* infection decreases the abundance of succinate-producing bacteria and succinate production, followed by the decreased levels of propionate, and thereby impairing gut barrier function and inducing anxiety.

It should be pointed out that other metabolites produced by gut microbiome may also have a role in the pathogenesis of *T. gondii*-induced anxiety-like behaviors. For example, significant reductions in raffinose and gluconic acid lactone were observed in the FMT-Tg group (Supplementary Figure S8d). It is reported that raffinose can regulate the balance of gut microbiota and protect epithelial integrity.^97,98^ Gluconic acid lactone is known as an oxidized derivative of glucose, and is capable of scavenging free radicals.^99^ These metabolites deserve more investigations in the future. Moreover, RNA sequencing or proteomics are needed to demonstrate the effects of antibiotics and succinate on brain function in the future, which may reveal previously unknown mechanistic insights that have not been considered before.

Previous studies have reported that cysts are an important basis for *T. gondii*-induced anxiety,^9^ which results from the preferential parasitization of the cysts in several key brain regions including the amygdala.^15^ We observed that Ab treatment of gut microbiota significantly decreased cyst burden in the brains of infected mice. This may be due to the direct inhibition of tachyzoite survival induced by high concentration of Ab cocktail. Thus, it is possible that Ab treatment ameliorates *T. gondii*-induced anxiety *via* decreasing cyst burden. However, in the FMT experiment, the only difference between the two groups of recipient mice (FMT-Con and FMT-Tg) resulted from the gut microbiota of donor mice (Con mice and *T. gondii-*infected mice). Moreover, we did not find the cysts in the brains of FMT-Tg mice, while these recipient mice exhibited behavioral and neuropathological alteration similar to the donor infected mice. Therefore, the anxiety-like behaviors observed in FMT-Tg mice should be associated with the gut microbiota of *T. gondii*-infected mice. However, in order to completely demonstrate if *T. gondii*-associated anxiety is dependent on gut microbiota, further experiments should be done in germ-free mice.

## Conclusion

The present study uncovers the role of the gut microbiota in mediating the anxiety-like behaviors induced by long-term infection of *T. gondii* in mice. Mechanistically, chronic *T. gondii* infection induces anxiety-like behaviors by disrupting intestinal homeostasis and altering gut microbiota to increase the neuroinflammation in the amygdala of mice. Intriguingly, chronic *T. gondii* infection decreases the abundance of succinate-producing bacteria, thereby decreasing the levels of intestinal succinate. Notably, we demonstrate that DBM can attenuate anxiety-like behaviors by restoring the intestinal barrier and immune homeostasis. Overall, these findings provide novel insights for understanding the pathogenesis of *T. gondii*-related anxiety.

## Supplementary Material

Supplemental Material

## Data Availability

The Transcriptome analysis data and 16S rRNA sequencing data in this study are available in the Sequence Read Archive (S.R.A.) under project numbers PRJNA950120 and PRJNA948817, respectively.
