# Supplementary material for: Gut microbiota mediates anxiety-like behaviors induced by chronic infection of Toxoplasma gondii in mice
Source: Gut Microbes. 2024 Aug 25;16(1):2391535. doi: 10.1080/19490976.2024.2391535 (PMC11346544; doi:10.1080/19490976.2024.2391535)
Supplement: Supplemental Material [file KGMI_A_2391535_SM6156.zip › Supplementary Material (1).docx]

**Gut microbiota mediates anxiety-like behaviors induced by chronic infection of *Toxoplasma gondii* in mice**

**Xiaotong Luo^1,2,3#^, Xiaoying Yang^1#^, Shimin Tan^1,2,3#^, Yongsheng Zhang^1,3#^, Yunqiu Liu^1#^, Xiaokang Tian^1,3^, Yingting Huang^1,2,3^, Yuying Zhou^1,3^, Cheng He^1^, Kun Yin^4^, Daxiang Xu^1^, Xiangyang Li^1^, Fenfen Sun^1,3^, Renxian Tang^1,3^, Jianping Cao^5^*, Kuiyang Zheng^1,3^*, Yinghua Yu^1^*, Wei Pan^1,3,5^***

**^#^These authors have contributed equally to this work**

**Affiliations**

^1^Jiangsu Key Laboratory of Immunity and Metabolism, Jiangsu International Key Laboratory of Immunity and Metabolism, Department of Pathogen Biology and Immunology, Xuzhou Medical University, Xuzhou, Jiangsu 221004, China;

^2^The Second Clinical Medical College, Xuzhou Medical University, Xuzhou, Jiangsu 221004, China;

^3^National Demonstration Center for Experimental Basic Medical Science Education, Xuzhou Medical University, Xuzhou, Jiangsu 221004, China；

^4^Shandong Institute of Parasitic Diseases, Shandong First Medical University & Shandong Academy of Medical Sciences, Jining, Shandong 272033, China;

^5^National Institute of Parasitic Diseases, Chinese Center for Disease Control and Prevention (Chinese Center for Tropical Diseases Research); NHC Key Laboratory of Parasite and Vector Biology; WHO Collaborating Centre for Tropical Diseases; National Center for International Research on Tropical Diseases; Shanghai 200025, China.

***Correspondence**

Wei Pan: [panwei525@126.com;](mailto:panwei525@126.com;) panwei@xzhmu.edu.cn.

Yinghua Yu: 3292965589@qq.com; yinghua@uow.edu.au.

Kuiyang Zheng: zky02@163.com.

Jianping Cao: caojpcdc@163.com.


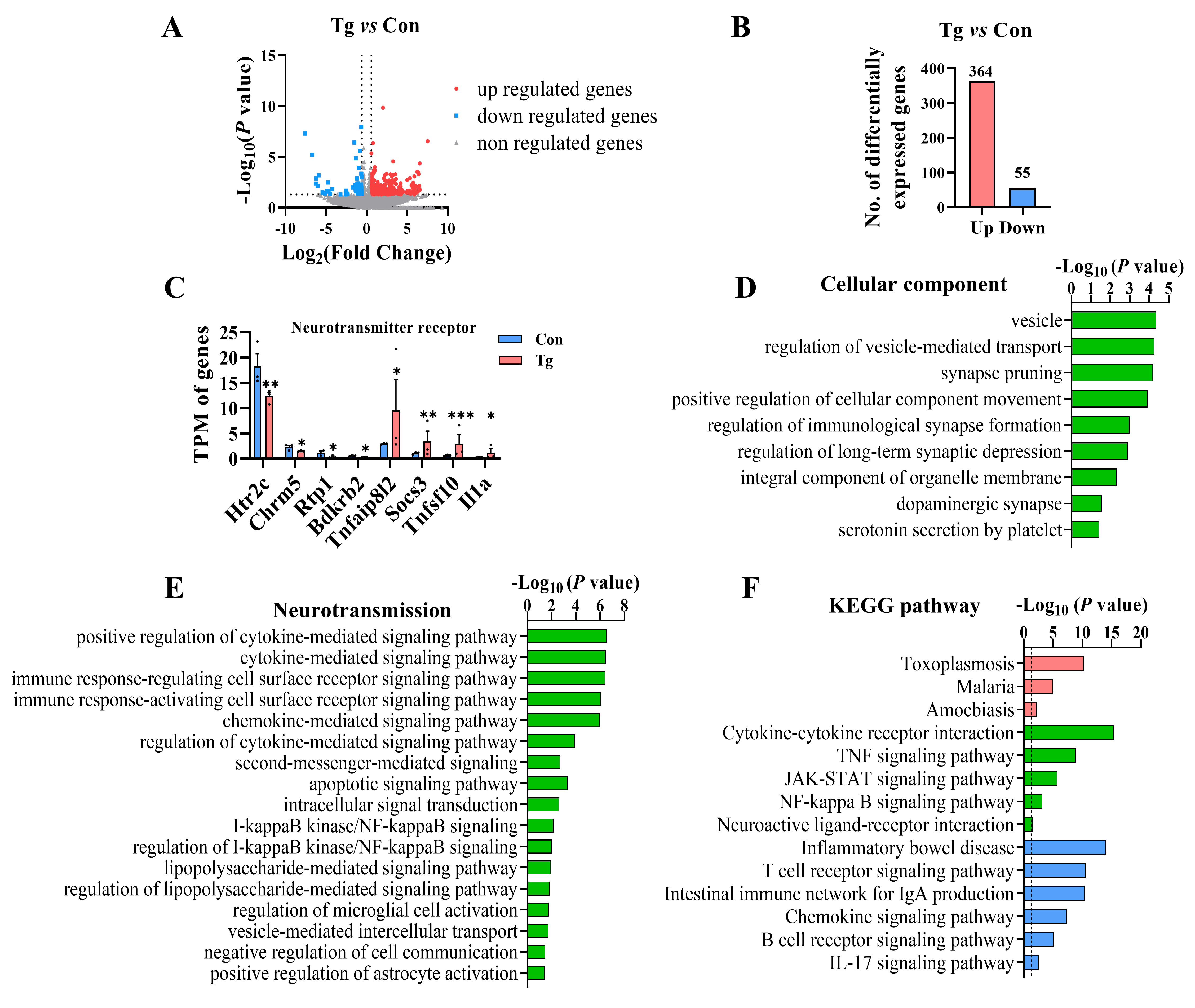


**Supplementary Figure 1. Chronic *T. gondii* infection alters the transcriptomic profile associated with anxiety in the amygdala of mice.** The transcriptomic profile of the amygdala was determined by RNA sequencing (*n* = 3). **A** The volcano plot shows the distributions of differentially expressed genes (DEGs) between the Con and Tg mice. **B** The number of upregulated and downregulated DEGs. **C** Normalized expression of selected genes regulating neurotransmitter receptor. **D** The biological processes associated with cellular component are significantly enriched in the Tg group. **E** The biological processes associated with neurotransmission are significantly enriched in the Tg group. **F** The enriched KEGG pathways. Columns with different colors represent different classifications at level 2. The dotted line in the figure represents *P* = 0.05. Values are presented as mean ± SEM. ^*^*P* < 0.05, ^**^*P* < 0.01, ^***^*P* < 0.001.

**
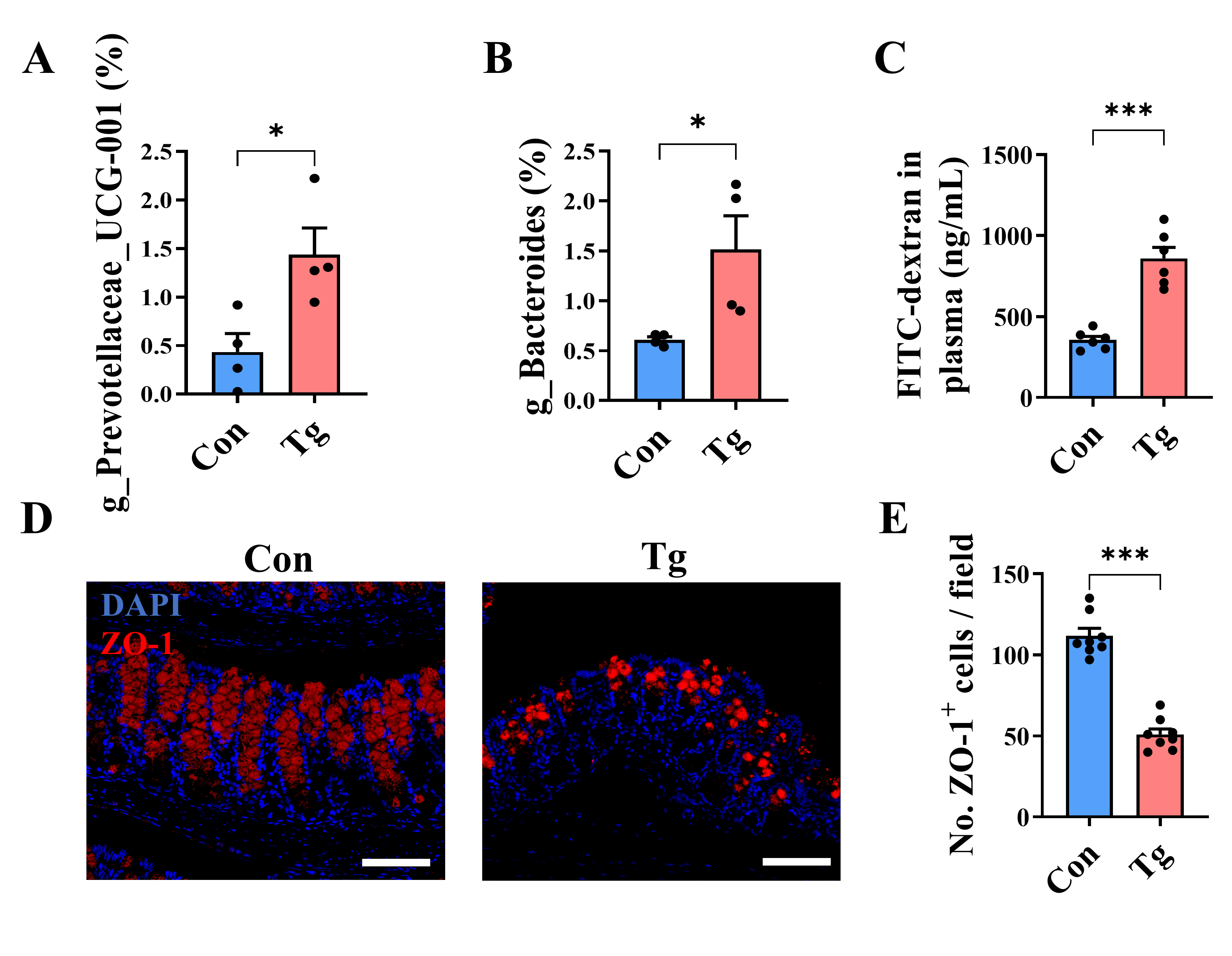
**

**Supplementary Figure 2.** **Chronic *T. gondii* infection impairs the colonic barrier integrity in mice.** Fecal microbiome composition was analyzed by 16S rRNA gene sequencing. **A** Relative abundance of *Prevotellaceae_UCG-001*. **B** Relative abundance of *Bacteroides*. **C** Intestinal permeability measured by FITC-dextran assay (*n* = 6). **D** Representative Immunofluorescence images of colonic sections stained with anti-ZO-1 antibody. Scale bar: 100 μm. **E** Number of ZO-1^+^ cells per field. Values are mean ± SEM. ^*^*P* < 0.05, ^***^*P* < 0.001.


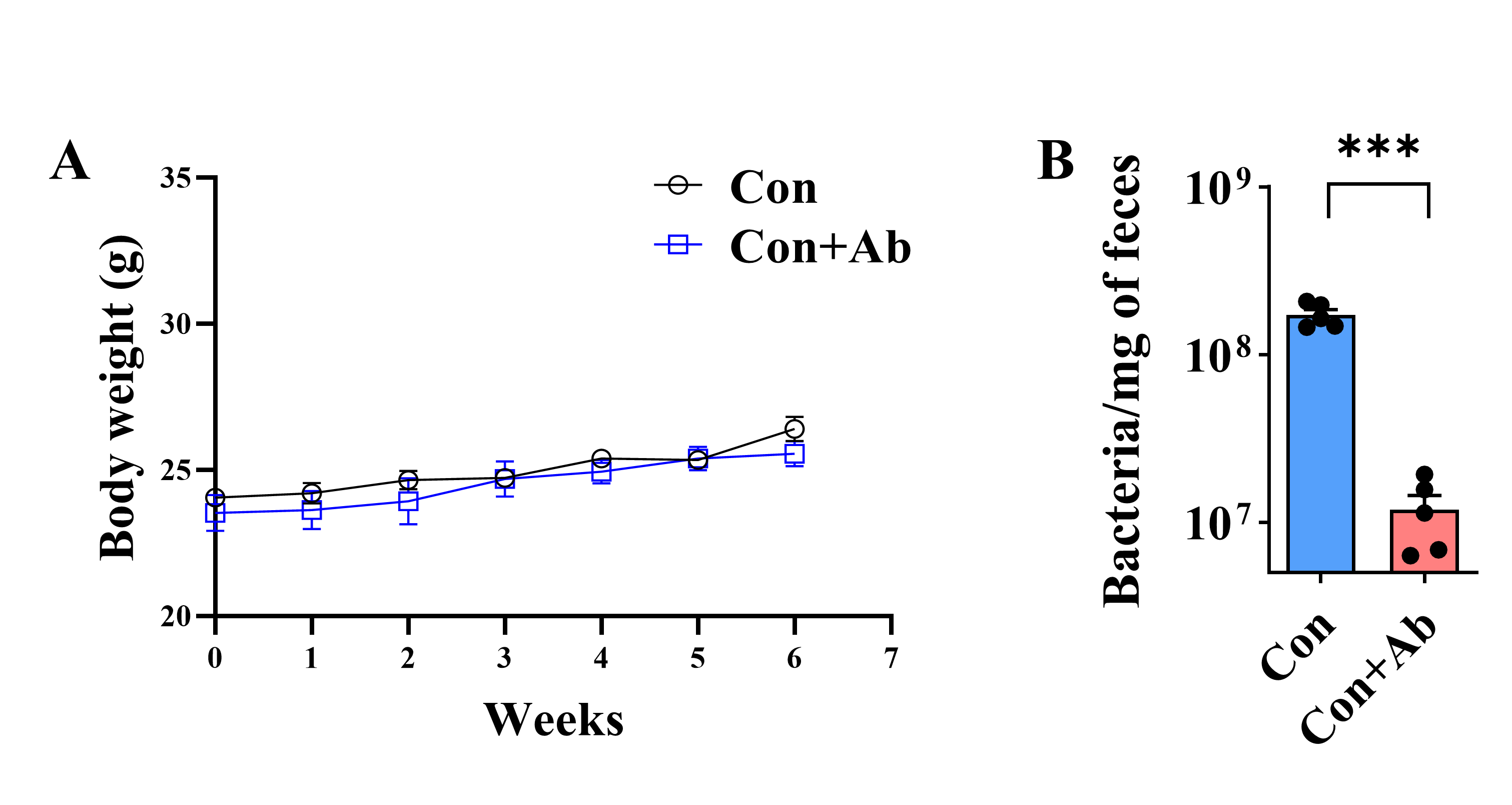


**Supplementary Figure 3. The effects of antibiotic cocktail on body weight and bacteria load of C57BL/6J mice. A** Body weight change of mice treated with antibiotic cocktail in drinking water for six week. **B** Levels of bacterial DNA in the feces of mice treated with antibiotic cocktail at the third week. Con: control group; Con+Ab: the mice treated with antibiotic cocktail. Values are mean ± SEM. Values are mean ± SEM. ^***^*P* < 0.001.

**
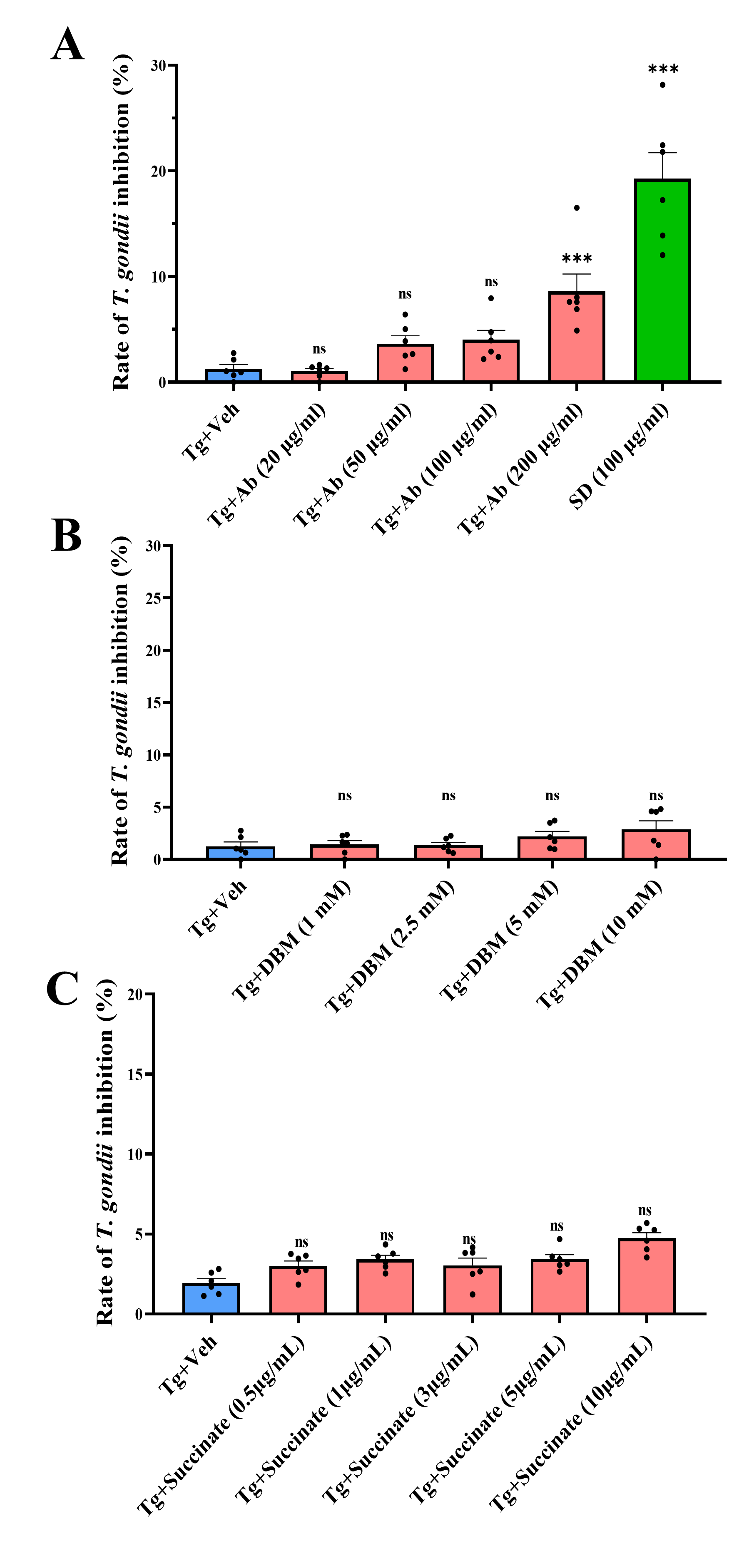
**

**Supplementary Figure 4. Inhibitory effects of** **antibiotics cocktail, DBM and succinate on *T. gondii* tachyzoites.** **A** Inhibition rates of antibiotics cocktail or SD treatment on tachyzoites growth (*n* = 6). **B** Inhibition rates DBM treatment on tachyzoites growth (*n* = 6). **C** Inhibition rates of succinate treatment on tachyzoites (*n* = 6). Tg+Veh: tachyzoites treated with vehicle control (PBS); Tg+Ab: tachyzoites treated with antibiotics cocktail; Tg+DBM: tachyzoites treated with DBM; Tg+Succinate: tachyzoites treated with succinate. SD: sulfadiazine. DBM: Diethyl butylmalonate. Values are presented as mean ± SEM. ^***^*P* < 0.001.


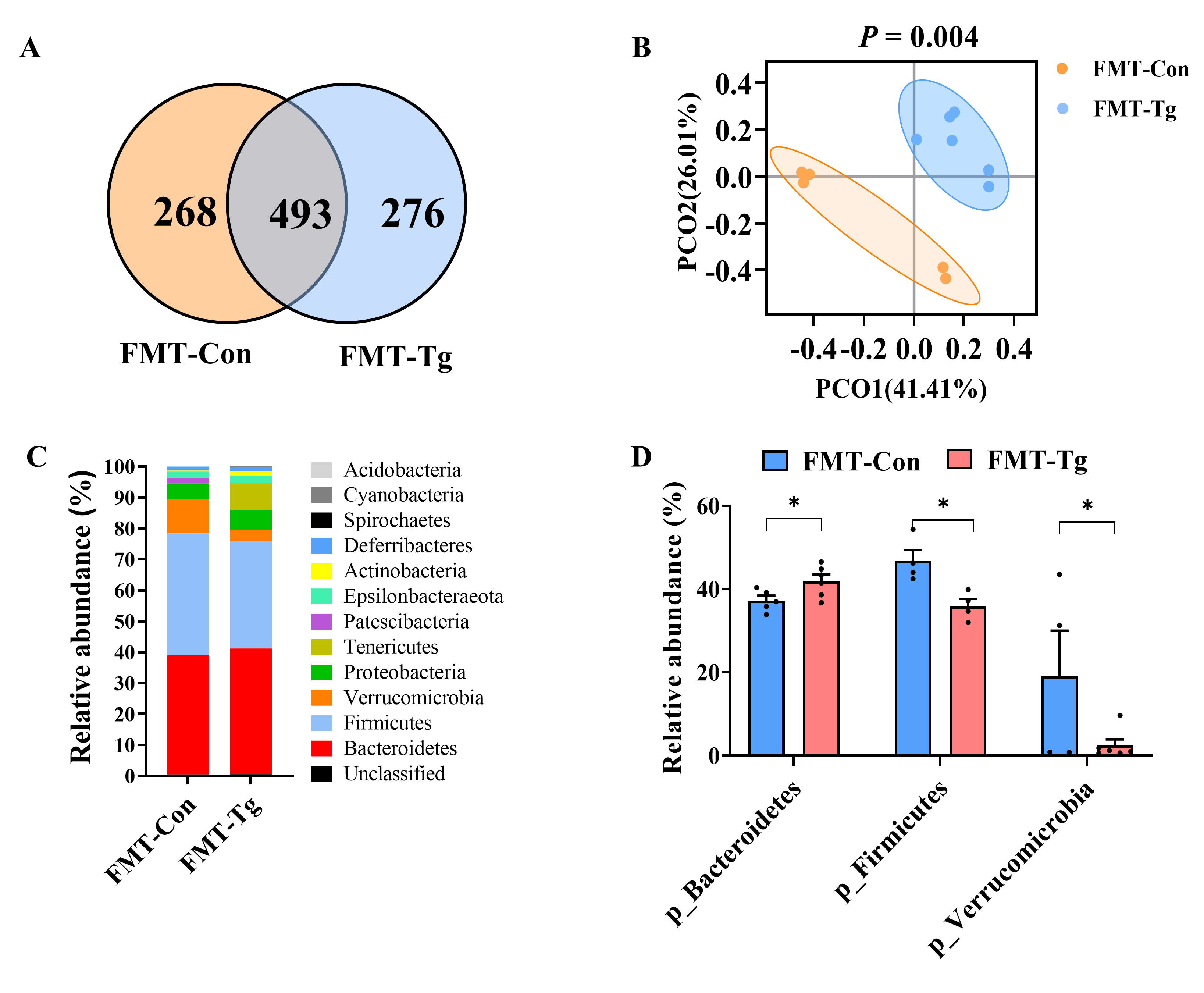


**Supplementary Figure 5. The microbiome composition of mice receiving fecal microbiome transplantation.** The microbiome composition in the fecal of the recipient mice post fecal microbiome transplantation was analyzed by 16S rRNA gene sequencing. **A** The number of shared and unique core OTUs between the FMT-Con and FMT-Tg groups. **B** Principal co-ordinate analysis of Bray distance (*P*-value from Anosim analysis are shown). **C** Composition abundance of bacterial phylum in the FMT-Con and FMT-Tg groups. **D** Comparison of the representative taxonomic abundance among FMT-Con and FMT-Tg groups.Values are presented as mean ± SEM. ^*^*P* < 0.05.


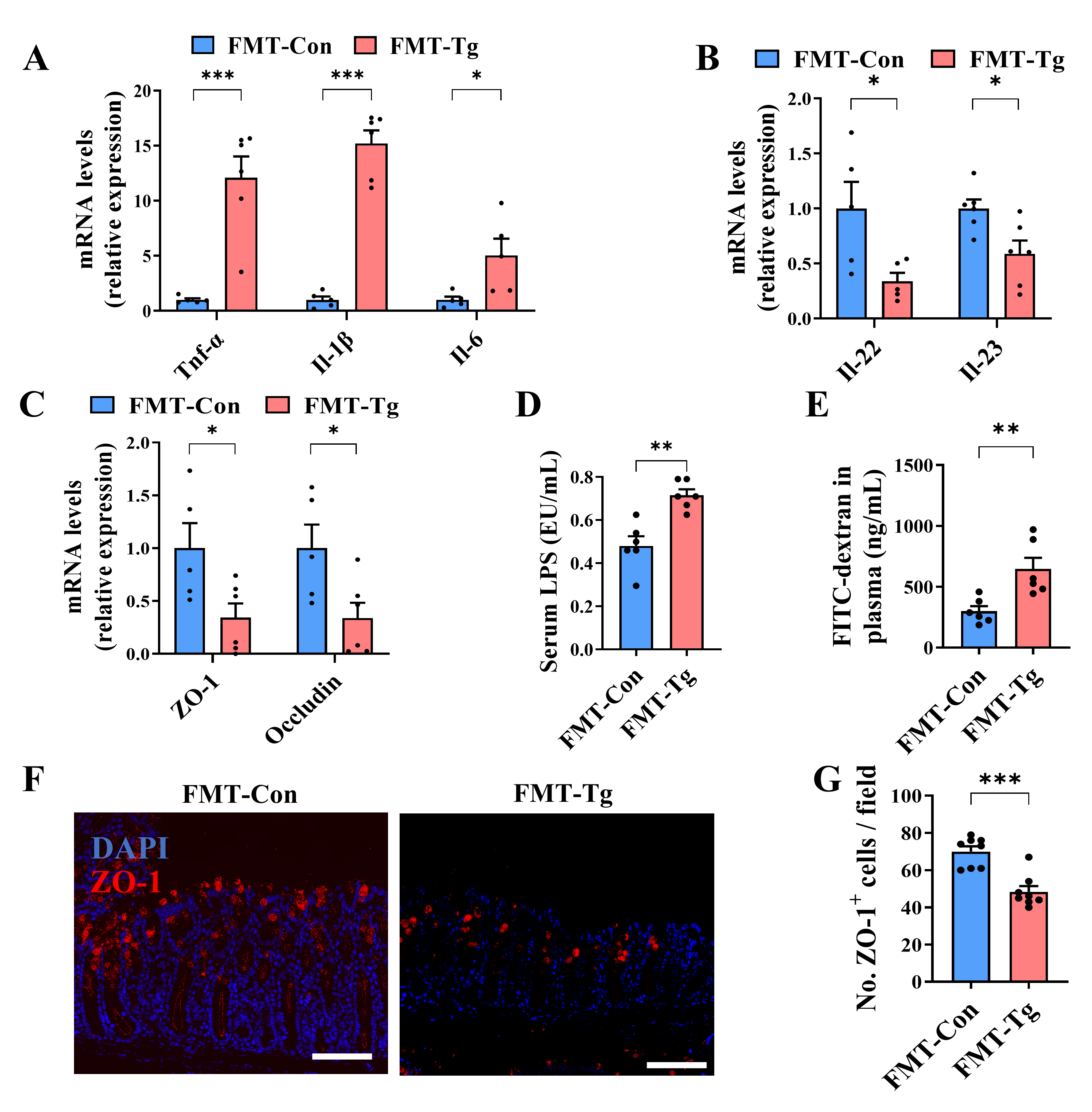


**Supplementary Figure 6.** **Transplantation of the fecal microbiota from *T. gondii* infected mice induces colonic inflammation and impairs gut barrier integrity in the recipient mice pretreated with antibiotic cocktail.** **A** mRNA expression of *Tnf-α*, *Il-1β*, *Il-6* in the colon (*n* = 4~6). **B** mRNA expression of *Il-22*, *Il-23* in the colon (*n* = 4~6). **C** mRNA expression of ZO-1, occludin in the colon (*n* = 4~6). **D** Level of LPS in the serum (*n* = 6). **E** Intestinal permeability measured by FITC-dextran assay (*n* = 6). **F** Representative Immunofluorescence images of colonic sections stained with anti-ZO-1 antibody. Scale bar: 100 μm. **G** Number of ZO-1^+^ cells per field. FMT-Con: the mice were firstly given antibiotics for three weeks and then transplanted fecal microbiota from Con mice; FMT-Tg: the mice were firstly given antibiotics for three weeks and then transplanted fecal microbiota from Tg-infected mice. Values are presented as mean ± SEM. ^*^*P* < 0.05, ^**^*P* < 0.01, ^***^*P* < 0.001.


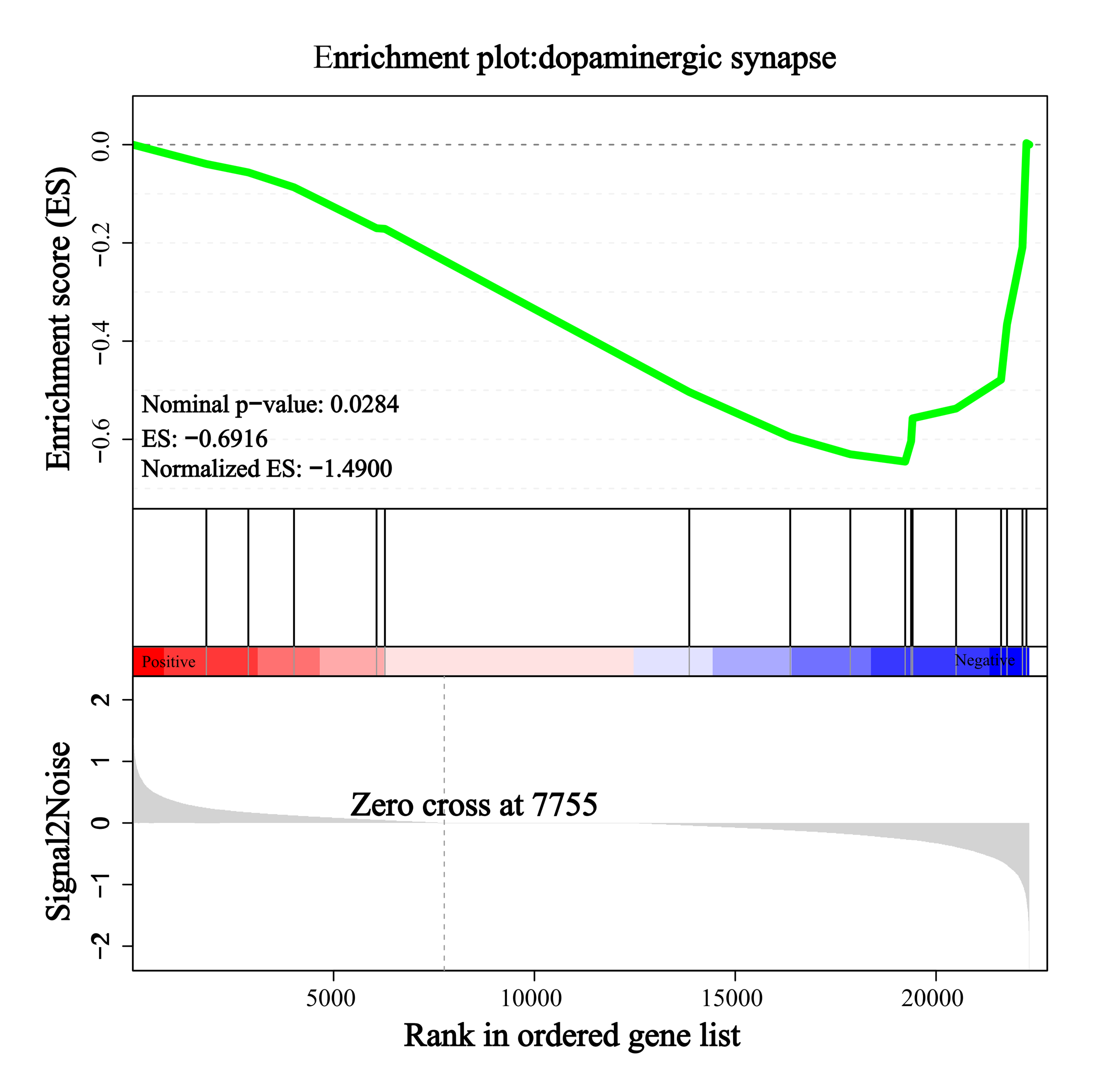


**Supplementary Figure 7.** **Identification of dopaminergic synapse pathway by gene Collection enrichment analysis (GSEA) in the amygdala of FMT-Tg mice.**


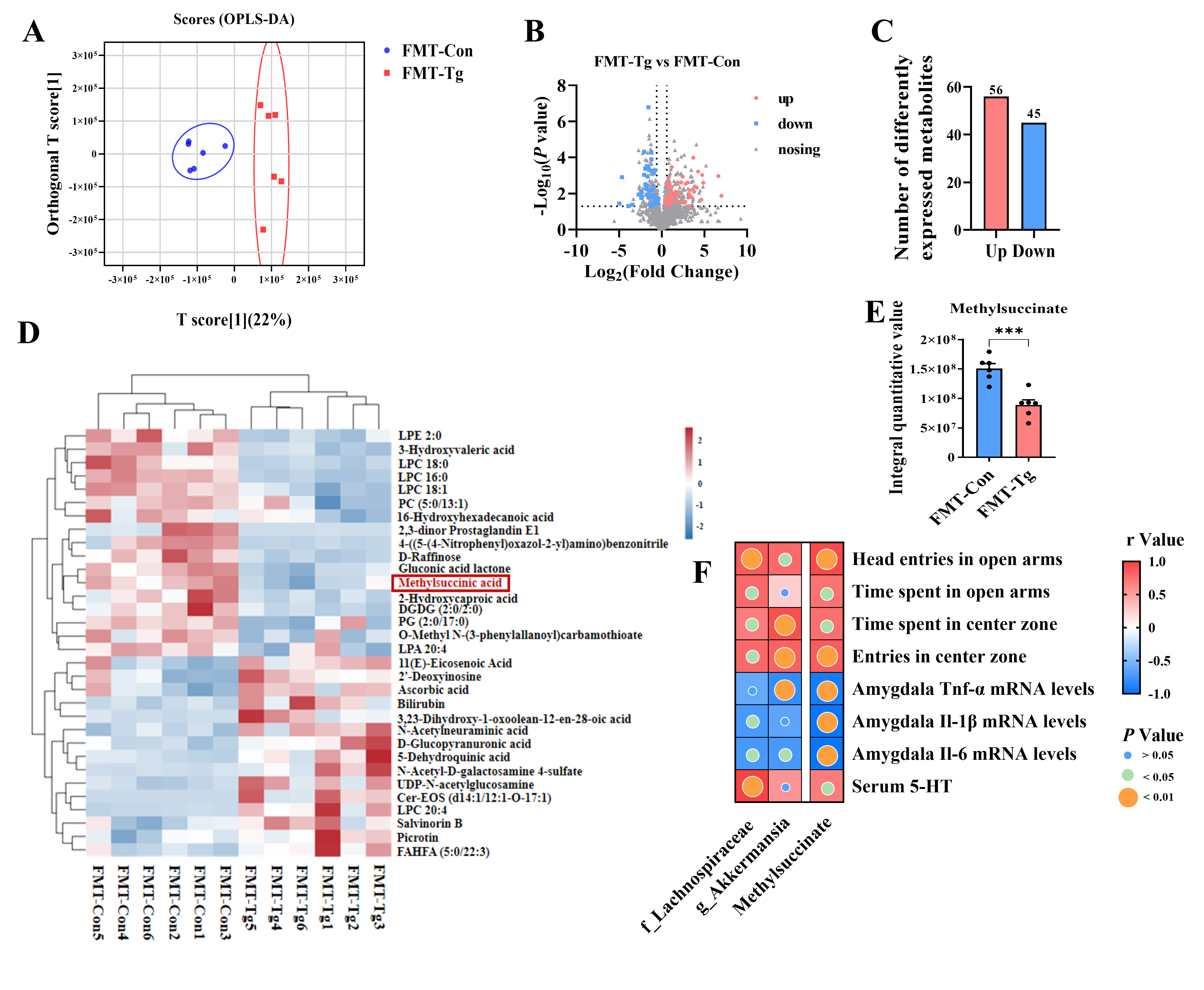


**Supplementary Figure 8.** **Transplantation of the fecal microbiota from *T. gondii* infected mice alters the metabolomic profile in the feces of of mice treated with antibiotics cocktails. A** Score chart of OPLS-DA. **B** Volcano map of differential metabolites. **C** The number of differential metabolites expressed by bar-chart. **D** Heat map of differential metabolite clustering. **E** Integral quantitative value of methylsuccinate. **F** The association between intestinal succinate and neuroinflammation, anxiety-like behaviors in the FMT-Con and FMT-Tg groups. *n* = 6. Values are presented as mean ± SEM. ^***^*P* < 0.001.


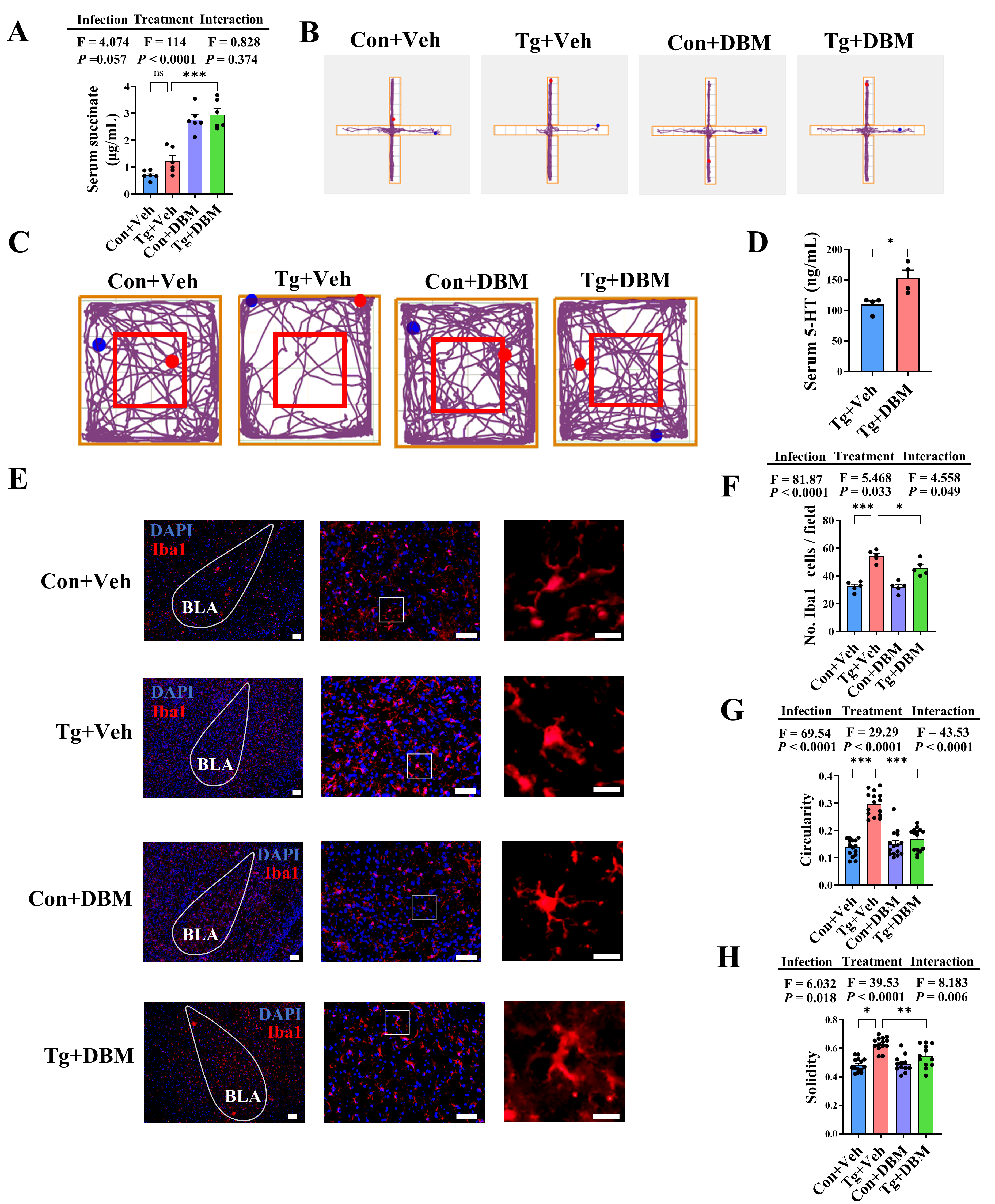


**Supplementary Figure 9. DBM supplementation improves the anxiety-like behaviors and neuroinflammation induced by** **chronic *T. gondii* infection. A** Effect of DBM on the succinate levels in serum (*n* = 6). **B** Representative figures of movement traces in the EPMT. **C** Representative figures of movement traces in the OFT. **D** Level of 5-HT in the serum (*n* = 4). **E** Immunofluorescence staining for Iba1 in the amygdala (scale bar 200 μm). The image was captured from the box (scale bar 50 μm). **F** Quantification of Iba1^+^ microglia in the amygdala. Cell circularity (**G**) and solidity (**H**) were used to show the microglial morphology. Con+Veh: [control group](javascript:;) treated with PBS; Tg+Veh: the mice infected with *T. gondii* cysts and treated with PBS; Con+DBM: the Con mice treated with DBM; Tg+DBM: the mice infected with *T. gondii* cysts and treated with DBM. DBM: Diethyl butylmalonate. *n* = 8. Values are presented as mean ± SEM. ^*^*P* < 0.05, ^**^*P* < 0.01, ^***^*P* < 0.001.


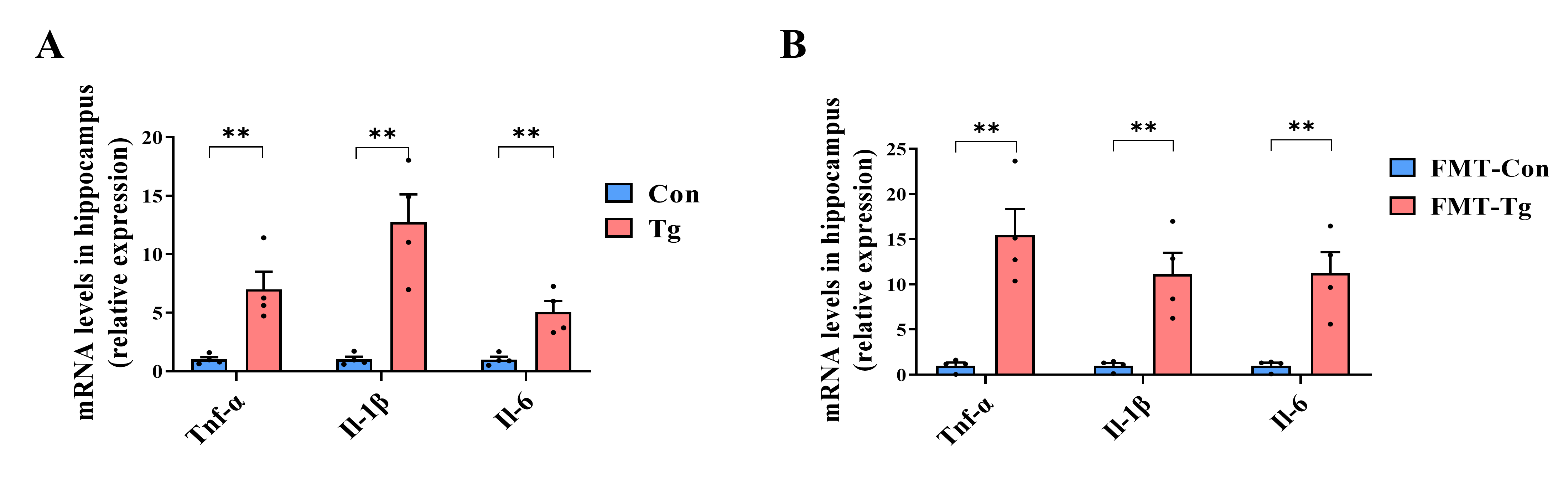


**Supplementary Figure 10. The upregulated expression of pro-inflammatory cytokines in the hippocampus of *T. gondii* infected mice and FMT-Tg mice. A** mRNA expression of *Tnf-α*, *Il-1β*, *Il-6* in the hippocampus in the Con and Tg groups (*n* = 4). **B** mRNA expression of *Tnf-α*, *Il-1β*, *Il-6* in the hippocampus in the FMT-Con and FMT-Tg groups (*n* = 4). Values are presented as mean ± SEM. ^**^*P* < 0.01.


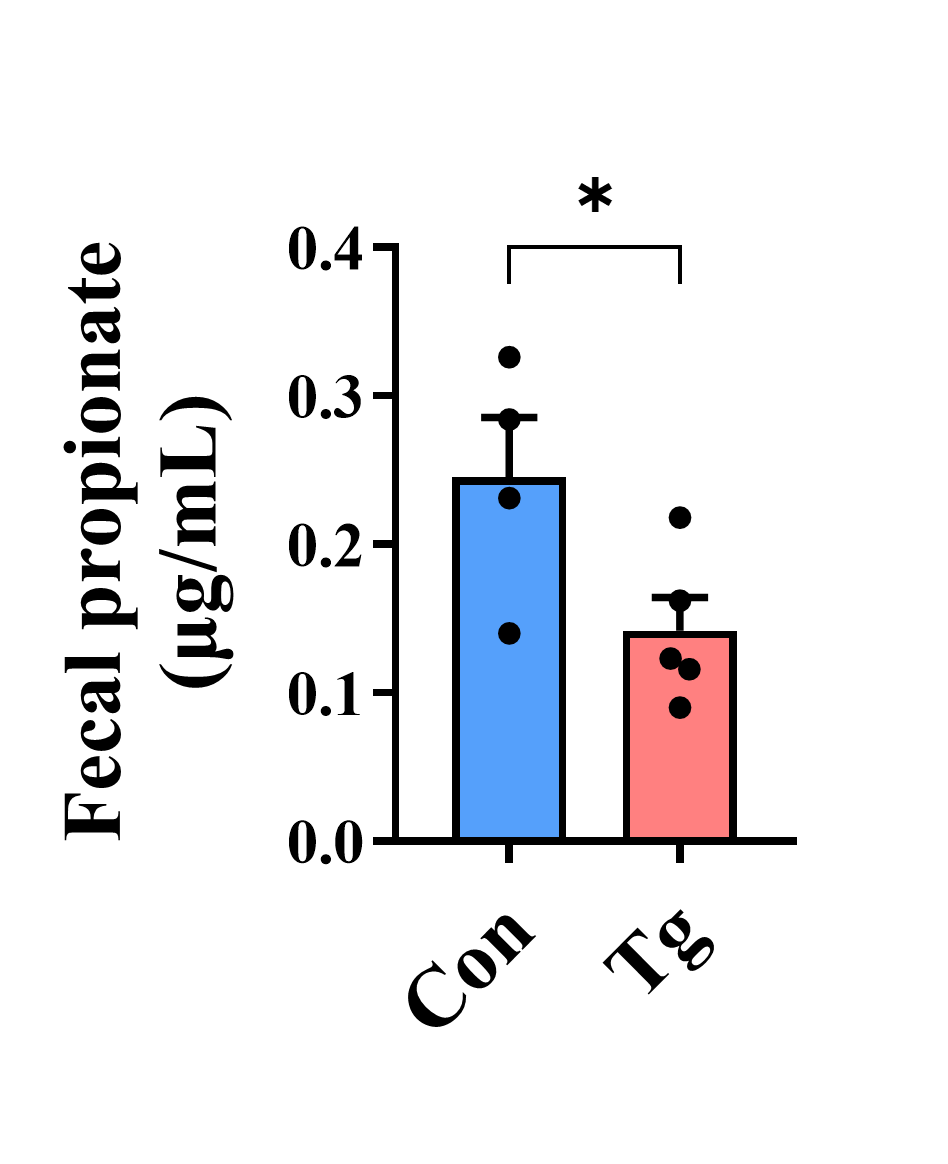


**Supplementary Figure 11.** ***T. gondii* chronic infection decreases the level of propionate in the feces of mice.** Values are presented as mean ± SEM. *^*^P* < 0.05.

**Supplementary Table 1. The qRT-PCR primer sequences used in this study**

| Num | Gene Symbol | Forward primer (5’-3’) | Reverse primer (5’-3’) |
| --- | --- | --- | --- |
| **1** | β-actin | AGAAGGTGGTGAAGCAGGCATC | CGAAGGTGGAAGAGTGGGAGTTG |
| **2** | *Il-22* | GCCAGCCTTGCAGATAACAA | GTTTGGTCAGGAAAGGCACC |
| **3** | *Il-23* | CAGCAGCTCTCTCGGAATCTC | TGGATACGGGGCACATTATTTTT |
| **4** | *Il-1β* | TGGGAAACAACAGTGGTCAGG | CTGCTCATTCACGAAAAGGGA |
| **5** | *Il-6* | TCACAGAAGGAGTGGCTAAGGACC | ACGCACTAGGTTTGCCGAGTAGAT |
| **6** | *Tnf-α* | CTTGTTGCCTCCTCTTTTGCTTA | CTTTATTTCTCTCAATGACCCGTAG |
| **7** | ZO-1 | AATGAGGATGAGGTTGTGTC | TTGTAGTTGTGAAGAGATGGTG |
| **8** | Occludin | CTATGGGACAGGGCTCTTTGGA | AGGAAGCGATGAAGCAGAAGGC |
| **9** | *Cxcl-1* | ACTGCACCCAAACCGAAGTC | TGGGGACACCTTTTAGCATCTT |
| **10** | *Cxcl-10* | CCAAGTGCTGCCGTCATTTTC | GGCTCGCAGGGATGATTTCAA |
